# Differential gene expression analysis for multi-subject single-cell RNA-sequencing studies with *aggregateBioVar*

**DOI:** 10.1093/bioinformatics/btab337

**Published:** 2021-05-10

**Authors:** Andrew L Thurman, Jason A Ratcliff, Michael S Chimenti, Alejandro A Pezzulo

**Affiliations:** Department of Internal Medicine, Roy J. and Lucille A. Carver College of Medicine, University of Iowa, Iowa City, IA 52242, USA; Iowa Institute of Human Genetics, Roy J. and Lucille A. Carver College of Medicine, University of Iowa, Iowa City, IA 52242, USA; Iowa Institute of Human Genetics, Roy J. and Lucille A. Carver College of Medicine, University of Iowa, Iowa City, IA 52242, USA; Department of Internal Medicine, Roy J. and Lucille A. Carver College of Medicine, University of Iowa, Iowa City, IA 52242, USA

## Abstract

**Motivation:**

Single-cell RNA-sequencing (scRNA-seq) provides more granular biological information than bulk RNA-sequencing; bulk RNA sequencing remains popular due to lower costs which allows processing more biological replicates and design more powerful studies. As scRNA-seq costs have decreased, collecting data from more than one biological replicate has become more feasible, but careful modeling of different layers of biological variation remains challenging for many users. Here, we propose a statistical model for scRNA-seq gene counts, describe a simple method for estimating model parameters and show that failing to account for additional biological variation in scRNA-seq studies can inflate false discovery rates (FDRs) of statistical tests.

**Results:**

First, in a simulation study, we show that when the gene expression distribution of a population of cells varies between subjects, a naïve approach to differential expression analysis will inflate the FDR. We then compare multiple differential expression testing methods on scRNA-seq datasets from human samples and from animal models. These analyses suggest that a naïve approach to differential expression testing could lead to many false discoveries; in contrast, an approach based on pseudobulk counts has better FDR control.

**Availability and implementation:**

A software package, *aggregateBioVar*, is freely available on Bioconductor (https://www.bioconductor.org/packages/release/bioc/html/aggregateBioVar.html) to accommodate compatibility with upstream and downstream methods in scRNA-seq data analysis pipelines.

**Supplementary information:**

Raw gene-by-cell count matrices for pig scRNA-seq data are available as GEO accession GSE150211. Supplementary data are available at *Bioinformatics* online.

## 1 Introduction

Single-cell RNA-sequencing (scRNA-seq) enables analysis of the effects of different conditions or perturbations on specific cell types or cellular states. Multiple methods and bioinformatic tools exist for initial scRNA-seq data processing, including normalization, dimensionality reduction, visualization, cell type identification, lineage relationships and differential gene expression (DGE) analysis (Chen *et al.*, 2019; Hwang *et al.*, 2018; Luecken and Theis, 2019; Vieth *et al.*, 2019; Zaragosi *et al.*, 2020).

A common use of DGE analysis for scRNA-seq data is to perform comparisons between pre-defined subsets of cells (referred to here as *marker detection methods*); many methods have been developed to perform this analysis (Butler *et al.*, 2018; Delmans and Hemberg, 2016; Finak *et al.*, 2015; Guo *et al.*, 2015; Kharchenko *et al.*, 2014; Korthauer *et al.*, 2016; Miao *et al.*, 2018; Qiu *et al.*, 2017a, b; Wang *et al.*, 2019; Wang and Nabavi, 2018). Marker detection methods allow quantification of variation between cells and exploration of expression heterogeneity within tissues. In scRNA-seq studies, where cells are collected from multiple subjects (e.g. healthy versus disease), an additional layer of variability is introduced. DGE methods to address this additional complexity, which have been referred to as differential state (DS) analysis are just being explored in the scRNA-seq field (Crowell *et al.*, 2020; Lun *et al.*, 2016; McCarthy *et al.*, 2017; Van den Berge *et al.*, 2019; Zimmerman *et al.*, 2021).

In recent years, the reagent and effort costs of scRNA-seq have decreased dramatically as novel techniques have been developed (Aicher *et al.*, 2019; Briggs *et al.*, 2018; Cao *et al.*, 2017; Chen *et al.*, 2019; Gehring *et al.*, 2020; Gierahn *et al.*, 2017; Klein *et al.*, 2015; Macosko *et al.*, 2015; Natarajan *et al.*, 2019; Rosenberg *et al.*, 2018; Vitak *et al.*, 2017; Zhang *et al.*, 2019; Ziegenhain *et al.*, 2017), so that *biological replication*, meaning data collected from multiple independent biological units such as different research animals or human subjects, is becoming more feasible; biological replication allows generalization of results to the population from which the sample was drawn. However, in studies with biological replication, gene expression is influenced by both cell-specific and subject-specific effects. Therefore, as experiments that include biological replication become more common, statistical frameworks to account for multiple sources of biological variability will be critical, as recently described by Lähnemann *et al.* (Lahnemann *et al.*, 2020).

Crowell *et al.* (Crowell *et al.*, 2020) provides a thorough comparison of a variety of DGE methods for scRNA-seq with biological replicates including: (i) marker detection methods, (ii) *pseudobulk* methods, where gene counts are aggregated between cells from different biological samples and (iii) mixed models, where models for gene expression are adjusted for sample-specific or batch effects. This study found that generally pseudobulk methods and mixed models had better statistical characteristics than marker detection methods, in terms of detecting differentially expressed genes with well-controlled false discovery rates (FDRs), and pseudobulk methods had fast computation times. In another study, mixed models were found to be superior alternatives to both pseudobulk and marker detection methods (Zimmerman *et al.*, 2021). Marker detection methods were found to have unacceptable FDR due to *pseudoreplication bias*, in which cells from the same individual are correlated but treated as independent replicates, and pseudobulk methods were found to be too conservative, in the sense that too many differentially expressed genes were undiscovered. Alternatively, batch correction methods have been proposed to remove inter-individual differences prior to DS analysis, however, this increases type I error rates and disturbs the rank-order of results as explained in Zimmerman *et al.* (Zimmerman *et al.*, 2021).

Here, we introduce a mathematical framework for modeling different sources of biological variation introduced in scRNA-seq data, and we provide a mathematical justification for the use of pseudobulk methods for DS analysis. These methods provide interpretable results that generalize to a population of research subjects, account for important sources of biological and technical variability and provide adequate FDR control.

We proceed as follows. First, we present a statistical model linking differences in gene counts at the cellular level to four sources: (i) subject-specific factors (e.g. disease and intervention), (ii) variation between subjects, (iii) variation between cells within subjects and (iv) technical variation introduced by sampling RNA molecules, library preparation and sequencing. Second, we make a formal argument for the validity of a DS test with subjects as the units of analysis and discuss our development of a Bioconductor package that can be incorporated into scRNA-seq analysis workflows. Third, we examine properties of DS testing in practice, comparing cells versus subjects as units of analysis in a simulation study and using available scRNA-seq data from humans and pigs. Finally, we discuss potential shortcomings and future work.

## 2 Materials and methods

### 2.1 Statistical model

In bulk RNA-seq studies, gene counts are often assumed to follow a negative binomial distribution (Hardcastle and Kelly, 2010; Leng *et al.*, 2013; Love *et al.*, 2014; Robinson *et al.*, 2010). The negative binomial distribution has a convenient interpretation as a hierarchical model, which is particularly useful for sequencing studies. In the first stage of the hierarchy, gene expression for each sample is assumed to follow a gamma distribution with mean expression modeled as a function of sample-specific covariates. When samples correspond to different experimental subjects, the first stage characterizes biological variation in gene expression between subjects. In the second stage, the observed data for each gene, measured as a count, is assumed to follow a Poisson distribution with mean equal to the product of a size factor, such as sequencing depth, and gene expression generated in the first stage. The second stage represents technical variation introduced by the processes of sampling from a population of RNAs, building a cDNA library and sequencing. Increasing sequencing depth can reduce technical variation and achieve more precise expression estimates, and collecting samples from more subjects can increase power to detect differentially expressed genes.

In contrast, single-cell experiments contain an additional source of biological variation between cells. We propose an extension of the negative binomial model to scRNA-seq data by introducing an additional stage in the model hierarchy.

For clarity of exposition, we adopt and extend notations similar to (Love *et al.*, 2014). In a scRNA-seq experiment with multiple subjects, we assume that the observed data consist of gene counts for G genes drawn from multiple cells among n subjects. We also assume that cell types or states have been identified, DS analysis will be performed within each cell type of interest and henceforth, the notation corresponds to one cell type.

Define $K_{\mathrm{ijc}}$ to be the count for gene i in cell c collected from subject j, and a *size factor* $s_{jc}$ related to the amount of information collected from cell c in subject j (i=1,…G; $c=1,\ldots ,C_{j};j=1,\ldots ,n$). For example, a simple definition of $s_{jc}$ is the number of unique molecular identifiers (UMIs) collected from cell c of subject j. To measure heterogeneity in expression among different groups, we assume that mean expression for gene i in subject j is influenced by R subject-specific covariates $x_{j1},\;\ldots ,x_{jR}.$ Let $\mathrm{Gamma}\left(a,b\right)$ denote the gamma distribution with shape parameter a and scale parameter b, $\mathrm{Poisson}\left(m\right)$ denote the Poisson distribution with mean m and $\left[X|Y\right]$ denote the conditional distribution of random variable X given random variable Y. To characterize these sources of variation, we consider the following three-stage model:

- Expression of gene i in subject j follows a gamma distribution,
  $$\theta_{ij}∼\mathrm{Gamma}\left(\alpha_{i}^{-1},q_{ij}\alpha_{i}\right),$$
  where $\log\left(q_{ij}\right)=\sum_{r}x_{jr}\beta_{ir}$. The dispersion parameter $\alpha_{i}$ will be termed *subject-level variance*. The mean of $\theta_{ij}$ is $q_{ij}$, and its variance is $\alpha_{i}$.
- Given *subject-level expression* $\theta_{\mathrm{ij}}$, the *cell-level expression* in cell c, $\lambda_{\mathrm{ijc}}$, follows a gamma distribution,
  $$\left[\lambda_{\mathrm{ijc}}|\theta_{ij}\right]∼\mathrm{Gamma}\left(\sigma_{ij}^{-2},\theta_{ij}\sigma_{ij}^{2}\right).$$
  The parameter $\sigma_{ij}^{2}$ is termed the *cell-level variance* for subject j, which is allowed to vary between genes and subjects. The mean of $\lambda_{\mathrm{ijc}}$ is $\theta_{ij}$, and its variance is $\sigma_{ij}^{2}.$
- Conditional on the cell-level expression $\lambda_{\mathrm{ijc}}$, gene counts are modeled using a Poisson distribution,
  $$\left[K_{\mathrm{ijc}}|\lambda_{\mathrm{ijc}}\right]∼\mathrm{Poisson}\left(s_{jc}\lambda_{\mathrm{ijc}}\right).$$

In stage i, variation in expression between subjects is due to differences in covariates via the regression function $q_{ij}$ and residual subject-to-subject variation via the dispersion parameter $\alpha_{i}$. In stage ii, we assume that we have not measured cell-level covariates, so that variation in expression between cells of the same type occurs only through the dispersion parameter $\sigma_{ij}^{2}$. In stage iii, technical variation in counts is generated from a Poisson distribution. This model implicitly assumes that the only systematic variation in expression is due to subject-level covariates, and for a fixed level of covariates, any additional variation between subjects or cells is due to chance.

Although, in this work, we only consider the simple model presented above, the model could be extended to allow for systematic variation between cells by imposing a regression model in stage ii. If $z_{jc1},z_{jc2},\ldots ,z_{\mathrm{jcL}}$ are L cell-level covariates, then a log-linear regression model could take the form $\log\left(\theta_{\mathrm{ijc}}\right)=\sum_{l}z_{\mathrm{jcl}}\gamma_{\mathrm{ijl}}$.

### 2.2 Approximation for DS analysis

It is helpful to inspect the proposed model under a simplifying assumption. Suppose that cell-level variance $\sigma_{ij}^{2}\approx 0$. Under this assumption, $\lambda_{ij}\approx \theta_{ij}$ and the three-stage model reduces to a two-stage model. Define the *aggregated counts* $K_{ij}=\sum_{c}K_{\mathrm{ijc}}$, and let $s_{j}=\sum_{c}s_{jc}$. The marginal distribution of $K_{ij}$ is approximately negative binomial with mean $\mu_{ij}=s_{j}q_{ij}$ and variance $\mu_{ij}+\alpha_{i}\mu_{ij}^{2}$. This is the model used in DESeq2 (Love *et al.*, 2014).

In practice, this assumption is unlikely to be satisfied, but if we make modest assumptions about the growth rates of the size factors and numbers of cells per subject, we can obtain a useful approximation.

Theorem 1: The expected value of $K_{ij}$ is $\mu_{ij}=s_{j}q_{ij}$. Further, if we assume that, for some constants $k_{1}$ and $k_{2}$, $C_{j}^{-1}\sum_{c}s_{jc}\to k_{1}$ and $C_{j}^{-1}\sum_{c}s_{jc}^{2}\to k_{2}$ as $C_{j}\to \infty$, then the variance of $K_{ij}$ is $\mu_{ij}+\left\{\alpha_{i}+o\left(1\right)\right\}\mu_{ij}^{2}$.

Proof:

The expected value of $K_{ij}$ is computed by conditioning,

$${E(K}_{ij})\;=\;E\left[E\left(K_{ij}|\left\{\lambda_{\mathrm{ijc}},c=1,\ldots ,C_{j}\right\}\right)\right]$$

 

$$=\;E\left[E\left(\sum_{c}s_{jc}\lambda_{\mathrm{ijc}}|\theta_{ij}\right)\right]$$

 

$$=\;E\left[s_{j}\theta_{ij}\right]$$

 

$$=\;s_{j}q_{ij}$$

The variance of $K_{ij}$ is computed using iterative applications of the total variance formula (Ross, 2019),

$${\mathrm{Var}(K}_{ij})\;=\;\mathrm{Var}\left[E\left(K_{ij}|\left\{\lambda_{\mathrm{ijc}},c=1,\ldots ,C_{j}\right\}\right)\right]+E\left[\mathrm{Var}\left(K_{ij}|\left\{\lambda_{\mathrm{ijc}},c=1,\ldots ,C_{j}\right\}\right)\right]$$

 

$$=\;\mathrm{Var}\left[\sum_{c}s_{jc}\lambda_{\mathrm{ijc}}\right]+E\left[\sum_{c}s_{jc}\lambda_{\mathrm{ijc}}\right]$$

 

$$=\;\mathrm{Var}\left[E\left(\sum_{c}s_{jc}\lambda_{\mathrm{ijc}}|\theta_{ij}\right)\right]+E\left[\mathrm{Var}\left(\sum_{c}s_{jc}\lambda_{\mathrm{ijc}}|\theta_{ij}\right)\right]+s_{j}q_{ij}$$

 

$$=\;\mathrm{Var}\left[s_{j}\theta_{ij}\right]+E\left[\sigma_{ij}^{2}\theta_{ij}\sum_{c}s_{jc}^{2}\right]+s_{j}q_{ij}$$

 

$$=\;s_{j}^{2}q_{ij}^{2}\alpha_{i}+\sigma_{ij}^{2}\left(q_{ij}^{2}\alpha_{i}+q_{ij}^{2}\right)\left(\sum_{c}s_{jc}^{2}\right)+s_{j}q_{ij}$$

 

$$=\;s_{j}^{2}q_{ij}^{2}\left\{\alpha_{i}+\sigma_{ij}^{2}\left(1+\alpha_{i}\right)\left(\sum_{c}s_{jc}^{2}\right)/s_{j}^{2}\right\}+s_{j}q_{ij}$$

 

$$=\;s_{j}^{2}q_{ij}^{2}\left\{\alpha_{i}+\sigma_{ij}^{2}\left(1+\alpha_{i}\right)\left(C_{j}^{-1}\sum_{c}s_{jc}^{2}\right)C_{j}^{-1}/{\left(C_{j}^{-1}\sum_{c}s_{jc}\right)}^{2}\right\}+s_{j}q_{ij}$$

Applying the assumptions $C_{j}^{-1}\sum_{c}s_{jc}\to k_{1}$ and $C_{j}^{-1}\sum_{c}s_{jc}^{2}\to k_{2}$ completes the proof. ▪

To better illustrate the assumptions of the theorem, consider the case when the size factor $s_{jc}\;$is the same for all cells in a sample j and denote the common size factor as $s_{j}^{*}.$ In this case, $C_{j}^{-1}\sum_{c}s_{jc}=s_{j}^{*}$ and $C_{j}^{-1}\sum_{c}s_{jc}^{2}={\left(s_{j}^{*}\right)}^{2}$, and the theorem holds. The main idea of the theorem is that if gene counts are summed across cells and the number of cells grows large for each subject, the influence of cell-level variation on the summed counts is negligible.

Because these assumptions are difficult to validate in practice, we suggest following the guidelines for library complexity in bulk RNA-seq studies. Consider a purified cell type (PCT) study design, in which many cells from a cell type of interest could be isolated and profiled using bulk RNA-seq. The observed counts for the PCT study are analogous to the aggregated counts for one cell type in a scRNA-seq study. Furthermore, guidelines for library complexity in bulk RNA-seq studies apply to data with heterogeneity between cell types, so these recommendations should be sufficient for both PCT and scRNA-seq studies, in which data have been stratified by cell type.

Theorem 1 implies that when the number of cells per subject is large, the aggregated counts follow a distribution with the same mean and variance structure as the negative binomial model used in many software packages for DS analysis of bulk RNA-seq data.

### 2.3 Implementation via *aggregateBioVar*

Theorem 1 provides a straightforward approach to estimating regression coefficients $\beta_{i1},\ldots ,\beta_{iR}$, testing hypotheses and constructing confidence intervals that properly account for variation in gene expression between subjects. As an example, consider a simple design in which we compare gene expression for control and treated subjects. We set $x_{j1}=1$ for all j and define $x_{j2}$ as a dummy variable indicating that subject j belongs to the treated group. Then the regression model from Section 2.1 simplifies to $\log\left(q_{ij}\right)=\beta_{i1}+\beta_{i2}x_{j2}$. The null and alternative hypotheses for the i-th gene are $H_{0}^{\left(i\right)}:\beta_{i2}=0$ and $H_{0}^{\left(i\right)}:\beta_{i2}\neq 0$, respectively.

**Fig. 1.**
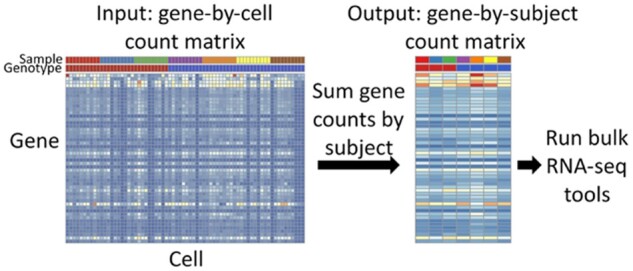
Aggregation technique accounting for subject-level variation in DS analysis. For each subject, gene counts are summed for all cells. The resulting matrix contains counts of each genefor each subject and can be analyzed using software for bulk RNA-seq data

We have developed the software package *aggregateBioVar* (available on Bioconductor) to facilitate broad adoption of pseudobulk-based DE testing; *aggregateBioVar* includes a detailed vignette, has low code complexity and minimal dependencies and is highly interoperable with existing RNA-seq analysis software using Bioconductor core data structures (Fig. 1). See Supplementary Material for brief example code demonstrating the usage of *aggregateBioVar*.

## 3 Results

In order to contrast DS analysis with cells as units of analysis versus subjects as units of analysis, we analysed both simulated and experimental data. In our simulation, the analysis focused on transcriptome-wide data simulated from the proposed model for scRNA-seq counts under different numbers of differentially expressed genes and different signal-to-noise ratios. To illustrate scalability and performance of various methods in real-world conditions, we show results in a porcine model of cystic fibrosis and analyses of skin, trachea and lung tissues in human sample datasets.

### 3.1 Simulation

We designed a simulation study to examine characteristics of using subjects or cells as units of analysis for DS testing under data simulated from the proposed model. Gene counts were simulated from the model in Section 2.1. To consider characteristics of a real dataset, we matched fixed quantities and parameters of the model to empirical values from a small airway secretory cell subset from the newborn pig data we present again in Section 3.2. Specifically, we considered a setting in which there were two groups of subjects to compare, containing four and three subjects, respectively with 21 731 genes. For each subject, the number of cells and numbers of UMIs per cell were matched to the pig data. The number of UMIs for cell c was taken to be the size factor $s_{jc}$ in stage 3 of the proposed model.

Further, the cell-level variance and subject-level variance parameters were matched to the pig data. Specifically, if $K_{\mathrm{ijc}}$ is the count of gene i in cell c from pig j, we defined $E_{\mathrm{ijc}}=K_{\mathrm{ijc}}/\sum_{i^{'}}K_{i^{'}jc}$ to be the normalized expression for cell c from subject j and $E_{ij}=\sum_{c}^{\;}K_{\mathrm{ijc}}/\sum_{i^{'}}^{\;}\sum_{c}K_{i^{'}jc}$ to be the normalized expression for subject j. Next, we matched the empirical moments of the distributions of $\left\{E_{ijc}\right\}$ and $\left\{E_{ij}\right\}$ to the population moments. If $m_{i}$ is the sample mean of {$E_{ij}$} over j, $v_{i}$ is the sample variance of {$E_{ij}$} over j, $m_{ij}$ is the sample mean of {$E_{\mathrm{ijc}}$} over c, and $v_{ij}$ is the sample variance of {$E_{\mathrm{ijc}}$} over c, we fixed the subject-level and cell-level variance parameters to be ${\tilde{\alpha }}_{i}=v_{i}/m_{i}^{2}$ and ${\tilde{\sigma }}_{ij}^{2}=v_{ij}/m_{ij}^{2}$, respectively.

The regression component of the model took the form $\log\left(q_{ij}\right)=\beta_{i1}+x_{j2}\beta_{i2}$, where $x_{j2}$ is an indicator that subject j is in group 2. The expression level of gene i for group 1, ${\tilde{\beta }}_{i1}$, was matched to the pig data by setting $e^{{\tilde{\beta }}_{i1}}=\sum_{j}^{\;}\sum_{c}^{\;}K_{\mathrm{ijc}}/\sum_{i^{'}}^{\;}\sum_{j}^{\;}\sum_{c}^{\;}K_{i^{'}jc}$. The expression parameter for the difference between groups 1 and 2, $\beta_{i2}$, was varied in order to evaluate the properties of DS analysis under a number of different scenarios.

Nine simulation settings were considered. First, a random proportion of genes, $p_{\mathrm{DE}}$, were flagged as differentially expressed. If a gene was not differentially expressed, the value of $\beta_{i2}$ was set to 0. If a gene was differentially expressed, $\beta_{i2}$ was simulated from a normal distribution with mean 0 and standard deviation (SD) τ. The value of $p_{\mathrm{DE}}$ describes the relative number of differentially expressed genes in a simulated dataset, and the value of τ controls the signal-to-noise ratio. As τ increases, the width of the distribution of effect sizes increases, so that the signal-to-noise ratio for differentially expressed genes is larger. We considered three values for $p_{\mathrm{DE}}\in \{0.01,\;0.3,\;0.6\}$, giving 1%, 30% and 60% of genes as differentially expressed, respectively, and we considered three values for τ∈{0.5, 1.0, 1.5}, representing low, medium and high signal-to-noise ratios, respectively. Comparisons of characteristics of the simulated and real data are shown in Supplementary Figures S1–S6.

For each setting, 100 datasets were simulated, and we compared seven different DS methods. The method *subject* treated subjects as the units of analysis, and statistical tests were performed according to the procedure outlined in Sections 2.2 and 2.3. The other six methods involved DS testing with cells as the units of analysis. Four of the methods were applications of the FindMarkers function in the R package Seurat (Butler *et al.*, 2018; Satija *et al.*, 2015; Stuart *et al.*, 2019) with different options for the type of test performed: for the method *wilcox*, cell counts were normalized, log-transformed and a Wilcoxon rank sum test was performed for each gene; for the method *NB*, cell counts were modeled using a negative binomial generalized linear model; for the method *MAST*, cell counts were modeled using a hurdle model based on the MAST software (Finak *et al.*, 2015) and for the method *DESeq2*, cell counts were modeled using the DESeq2 software (Love *et al.*, 2014). The other two methods were *Monocle*, which utilized a negative binomial generalized additive model to test for differences in gene expression using the R package Monocle (Qiu *et al.*, 2017a, b; Trapnell *et al.*, 2014) and *mixed*, which modeled counts using a negative binomial generalized linear mixed model with a random effect to account for differences in gene expression between subjects and DS testing was performed using a Wald test. For each method, the computed *P*-values for all genes were adjusted to control the FDR using the Benjamini–Hochberg procedure (Benjamini and Hochberg, 1995).

**Fig. 2.**
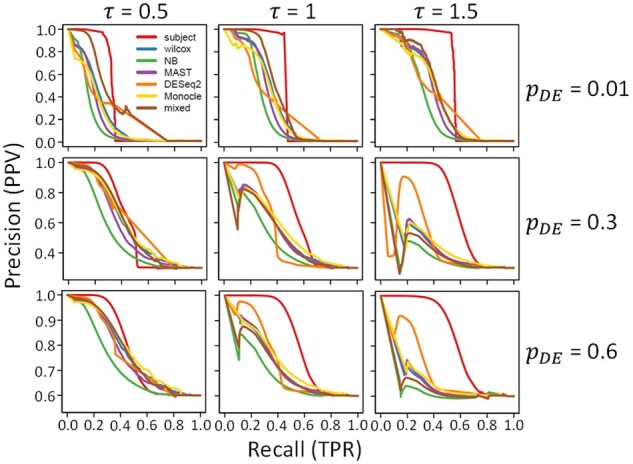
PR curves for DS analysis methods. Each panel shows results for 100 simulated datasets in one simulation setting. Rows correspond to different proportions of differentially expressed genes, $p_{\mathrm{DE}}$ and columns correspond to different SDs of (natural) log fold change, τ. In each panel, PR curves are plotted for each of seven DS analysis methods: subject (red), wilcox (blue), NB (green), MAST (purple), DESeq2 (orange), Monocle (gold) and mixed (brown). The vertical axis gives the precision (PPV) and the horizontal axis gives recall (TPR)

Figure 2 shows precision-recall (PR) curves averaged over 100 simulated datasets for each simulation setting and method. For a sequence of cutoff values between 0 and 1, precision, also known as positive predictive value (PPV), is the fraction of genes with adjusted *P*-values less than a cutoff (*detected genes*) that are differentially expressed. The recall, also known as the true positive rate (TPR), is the fraction of differentially expressed genes that are detected. According to this criterion, the *subject* method had the best performance, and the degree to which *subject* outperformed the other methods improved with larger values of the signal-to-noise ratio parameter τ.

**Fig. 3.**
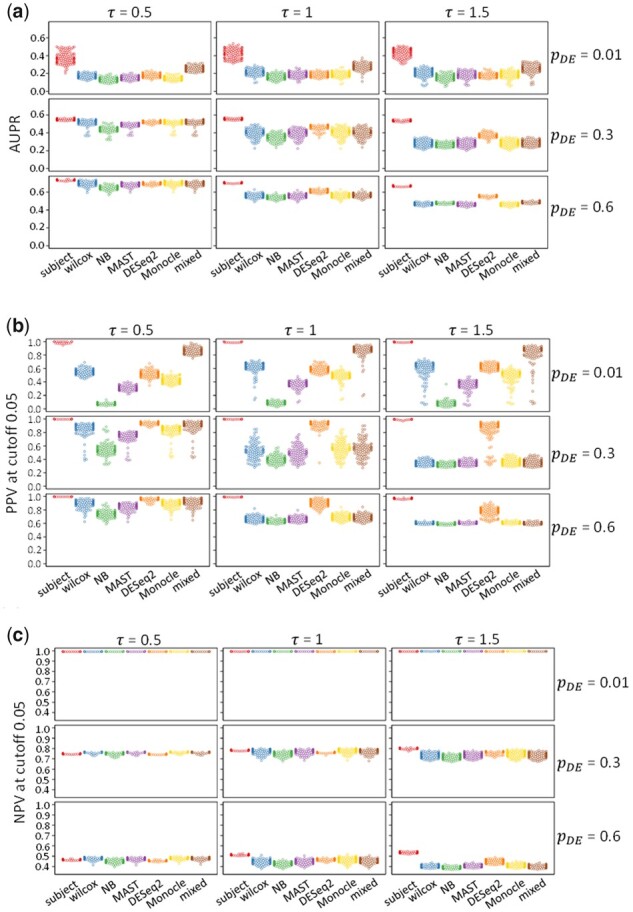
Performance measures for DS analysis of simulated data. (**a**) AUPR, (**b**) PPV with adjusted *P*-value cutoff 0.05 and (**c**) NPV with adjusted *P*-value cutoff 0.05 for 7 DS analysis methods. Each panel shows results for 100 simulated datasets in 1 simulation setting. Rows correspond to different proportions of differentially expressed genes, $p_{\mathrm{DE}}$ and columns correspond to different SDs of (natural) log fold change, τ. The implemented methods are subject (red), wilcox (blue), NB (green), MAST (purple), DESeq2 (orange), monocle (gold) and mixed (brown). The vertical axes give the performance measures, and the horizontal axes label each method

Figure 3a shows the area under the PR curve (AUPR) for each method and simulation setting. As we observed in Figure 2, the *subject* method had a larger area under the curve than the other six methods in all simulation settings, with larger differences for higher signal-to-noise ratios. When only 1% of genes were differentially expressed, the *mixed* method had a larger area under the curve than the other five methods.

In practice, often only one cutoff value for the adjusted *P*-value will be chosen to detect genes. Figure 3(b and c) show the PPV and negative predictive value (NPV) for each method and simulation setting under an adjusted *P*-value cutoff of 0.05. NPV is the fraction of undetected genes that were not differentially expressed.

The *subject* method had the highest PPV, and the *NB* method had the lowest PPV in all nine simulation settings. Among the other five methods, when the number of differentially expressed genes was small ($p_{\mathrm{DE}}$ = 0.01), the *mixed* method had the highest PPV values, whereas for higher numbers of differentially expressed genes ($p_{\mathrm{DE}}$ > 0.01), the *DESeq2* method had the highest PPV values. The *wilcox*, *MAST* and *Monocle* methods had intermediate performance in these nine settings.

Generally, the NPV values were more similar across methods. When only 1% of genes were differentially expressed ($p_{\mathrm{DE}}$ = 0.01), all methods had NPV values near 1. For higher numbers of differentially expressed genes ($p_{\mathrm{DE}}$ > 0.01), the *subject* method had lower NPV values when τ = 0.5 and similar or higher NPV values when τ > 0.5.

Results for alternative performance measures, including receiver operating characteristic (ROC) curves, TPRs and false positive rates (FPRs) can be found in Supplementary Figures S7 and S8. In general, the method *subject* had lower area under the ROC curve and lower TPR but with lower FPR.

The computations for each method were performed on the high-performance computing cluster at the University of Iowa. The cluster contains hundreds of computation nodes with varying numbers of processor cores and memory, but all jobs were submitted to the same job queue, ensuring that the relative computation times for these jobs were comparable. Supplementary Figure S9 contains computation times for each method and simulation setting for the 100 simulated datasets. The *subject* method had the shortest average computation times, typically <1 min. Four of the cell-level methods had somewhat longer average computation times, with *MAST* running for 7 min, *wilcox* and *Monocle* running for 9 min and *NB* running for 18 min. Two of the methods had much longer computation times with *DESeq2* running for 186 min and *mixed* running for 334 min.

### 3.2 DS analysis of airway epithelial secretory cells in a porcine model of cystic fibrosis

In addition to simulated data, we analysed an animal model dataset containing large and small airway epithelia from CF and non-CF pigs (Rogers *et al.*, 2008). Standard normalization, scaling, clustering and dimension reduction were performed using the R package Seurat version 3.1.1 (Butler *et al.*, 2018; Satija *et al.*, 2015; Stuart *et al.*, 2019). We identified cell types, and our DS analyses focused on comparing expression profiles between large and small airways and CF and non-CF pigs. Here, we present the DS results comparing CF and non-CF pigs only in secretory cells from the small airways. We performed DS analysis using the same seven methods as Section 3.1.

**Fig. 4.**
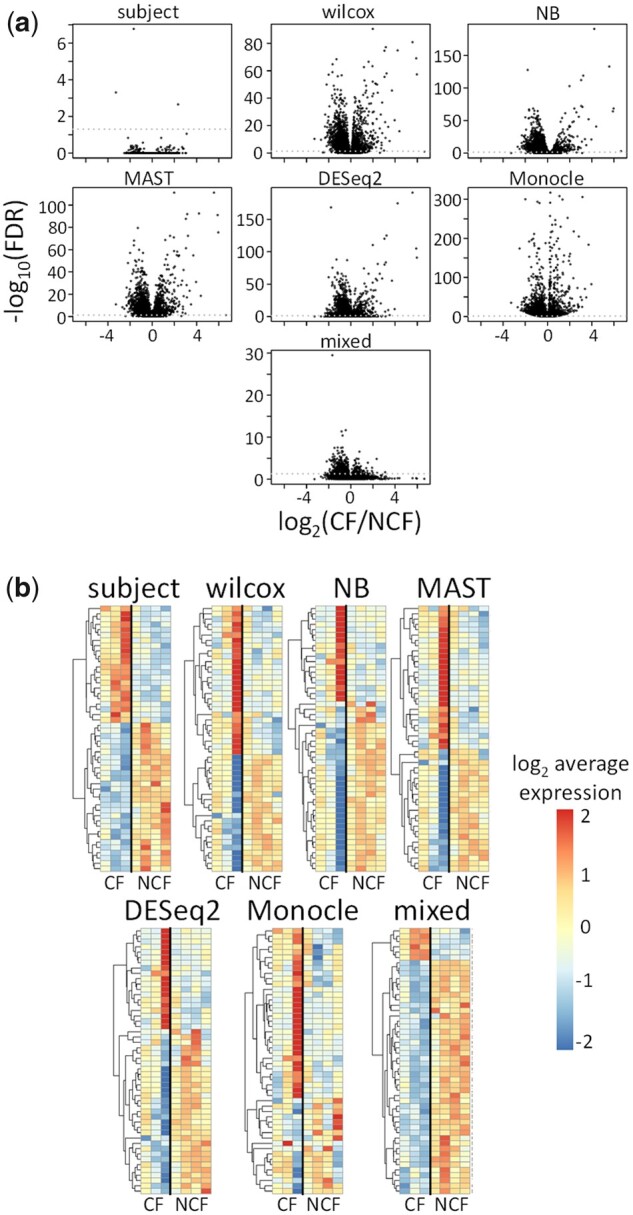
Results for analysis of CF and non-CF pig small airway secretory cells. (**a**) Volcano plots and (**b**) heatmaps of top 50 genes for 7 different DS analysis methods. In (a), vertical axes are negative log_10_-transformed adjusted *P*-values, and horizontal axes are log_2_-transformed fold changes. In (b), rows correspond to different genes, and columns correspond to different pigs. The top 50 genes for each method were defined to be the 50 genes with smallest adjusted *P*-values. Subject-level gene expression scores were computed as the average counts per million for all cells from each subject. NCF = non-CF

Figure 4a shows volcano plots summarizing the DS results for the seven methods. The volcano plot for the *subject* method shows three genes with adjusted *P*-value <0.05 (–log_10_(FDR) > 1.3), whereas the other six methods detected a much larger number of genes. The number of genes detected by *wilcox*, *NB*, *MAST*, *DESeq2*, *Monocle* and *mixed* were 6928, 7943, 7368, 4512, 5982 and 821, respectively. Among the three genes detected by *subject*, the genes *CFTR* and *CD36* were detected by all methods, whereas only *subject*, *wilcox*, *MAST* and *Monocle* detected *APOB*. Importantly, although these results specifically target differences in small airway secretory cells and are not directly comparable with other transcriptome studies, previous bulk RNA-seq (Bartlett *et al.*, 2016) and microarray (Stoltz *et al.*, 2010) studies have suggested few gene expression differences in airway epithelial tissues between CF and non-CF pigs; true differential gene expression between genotypes at birth is therefore likely to be small, as detected by the *subject* method.

Supplementary Figure S10 shows concordance between adjusted *P*-values for each method. These methods appear to form two clusters: the cell-level methods (*wilcox*, *NB*, *MAST*, *DESeq2* and *Monocle*) and the subject-level method (*subject*), with *mixed* sharing modest concordance with both clusters.

Figure 4b shows the top 50 genes for each method, defined by the smallest 50 adjusted *P*-values. The *subject* and *mixed* methods are composed of genes that have high inter-group (CF versus non-CF) and low intra-group (between subject) variability, whereas the *wilcox*, *NB*, *MAST*, *DESeq2* and *Monocle* methods tend to be sensitive to a highly variable gene expression pattern from the third CF pig.

In order to determine the reliability of the unadjusted *P*-values computed by each method, we compared them to the unadjusted *P*-values obtained from a permutation test. First, the CF and non-CF labels were permuted between subjects. For this study, there were 35 distinct permutations of CF and non-CF labels between the 7 pigs. Then, for each method, we defined the permutation test statistic to be the unadjusted *P*-value generated by the method. To obtain *permutation P-values*, we measured the proportion of permutation test statistics less than or equal to the observed test statistic, which is the permutation test statistic under the observed labels. For each method, we compared the permutation *P*-values to the *P*-values directly computed by each method, which we define as the *method P-values*.

Supplementary Figure S11 shows cumulative distribution functions (CDFs) of permutation *P*-values and method *P*-values. Because the permutation test is calibrated so that the permuted data represent sampling under the null distribution of no gene expression difference between CF and non-CF, agreement between the distributions of the permutation *P*-values and method *P*-values indicate appropriate calibration of type I error control for each method. Overall, the *subject* and *mixed* methods had the highest concordance between permutation and method *P*-values. Specifically, the CDFs are in high agreement for the *subject* method in the range of *P*-values from 0 to 0.2, whereas the *mixed* method has a slight inflation of small *P*-values in the same range compared to the permutation test. All of the other methods compute *P*-values that are much smaller than those computed by the permutation tests. These results suggest that only the *subject* method will exhibit appropriate type I error rate control. The lists of genes detected by the other six methods likely contain many false discoveries.

### 3.3 DS analysis of large and small airway ciliated cells in healthy pigs

Our analysis of CF and non-CF pigs showed that the *subject* method better controlled the FPR of DS analysis when the expected rate of true positives is small; here, using the same animal model, we compare large and small airway ciliated cells which are expected to vary largely.

Supplementary Figure S12a shows volcano plots for the results of the seven DS methods described. In this comparison, many genes were detected by all seven methods. We detected 6435, 13733, 12772, 13607, 13105, 14288 and 8318 genes by *subject*, *wilcox*, *NB*, *MAST*, *DESeq2*, *Monocle* and *mixed*, respectively. The volcano plots for *subject* and *mixed* show a stronger association between effect size (absolute log_2_-transformed fold change) and statistical significance (negative log_10_-transformed adjusted *P*-value).

Supplementary Figure S13 shows concordance between adjusted *P*-values for each method. If we omit *DESeq2*, which seems to be an outlier, the other six methods form two distinct clusters, with cluster 1 composed of *wilcox*, *NB*, *MAST* and *Monocle*, and cluster 2 composed of *subject* and *mixed*. The intra-cluster correlations are between 0.9 and 1, whereas the inter-cluster correlations are between 0.51 and 0.62. This figure suggests that the methods that account for between subject differences in gene expression (*subject* and *mixed*) will detect different sets of genes than the methods that treat cells as the units of analysis.

Supplementary Figure S12b shows the top 50 genes for each method, defined as the genes with the 50 smallest adjusted *P*-values. All seven methods identify two distinct groups of genes: those with higher average expression in large airways and those with higher average expression in small airways. The *subject* and *mixed* methods show the highest ratios of inter-group to intra-group variation in gene expression, whereas the other five methods have substantial intra-group variation. This suggests that methods that fail to account for between subject differences in gene expression are more sensitive to biological variation between subjects, leading to more false discoveries.

### 3.4 Marker detection for T cells and macrophages from human skin

Next, we applied our approach for marker detection and DS analysis to published human datasets. Given the similar performances of *wilcox*, *NB*, *MAST*, *DESeq2* and *Monocle*, in the simulations and animal model analysis, we only show the results for *subject*, *wilcox* and *mixed*. We performed marker detection analysis of cells obtained from a study of five human skin punch biopsies (Sole-Boldo *et al.*, 2020). Because we are comparing different cells from the same subjects, the *subject* and *mixed* methods can also account for the matching of cells by subject in the regression models.

Supplementary Figure S14 shows the results of marker detection for T cells and macrophages. For each of these two cell types, the expression profiles are compared to all other cells as in traditional marker detection analysis. Supplementary Figure S14(c–d) show that generally the shapes of the volcano plots are more similar between the *subject* and *mixed* methods than the *wilcox* method.

In Supplementary Figure S14(e–f), we quantify the ability of each method to correctly identify markers of T cells and macrophages from a database of known cell type markers (Franzen *et al.*, 2019). First, the adjusted *P*-values for each method are sorted from smallest to largest. Then, we consider the top *g* genes for each method, which are the *g* genes with the smallest adjusted *P*-values, and find what percentage of these top genes are known markers. For the T cells, (Supplementary Fig. S14e), we find that the *subject* and *wilcox* methods produce ranked gene lists with higher frequencies of marker genes than the *mixed* method, with *subject* having a slightly higher detection of known markers than *wilcox*. For macrophages (Supplementary Fig. S14f), *wilcox* produces better ranked gene lists of known markers than both *subject* and *wilcox* and again, the *mixed* method has the worst performance. Overall, these results suggest that the current marker detection analysis tools used in common practice, such as *wilcox*, will produce a reliable set of markers.

### 3.5 Marker detection for CD66+ and CD66- basal cells from human trachea

In order to objectively measure the performance of our tested approaches in scRNA-seq DS analysis, we compared them to a gold standard consistent of bulk RNA-seq analysis of purified/sorted cell types. In a scRNA-seq study of human tracheal epithelial cells from healthy subjects and subjects with idiopathic pulmonary fibrosis (IPF), the authors found that the basal cell population contained specialized subtypes (Carraro *et al.*, 2020). One such subtype, defined by expression of CD66, was further processed by sorting basal cells according to detection of CD66 and profiling by bulk RNA-seq. Here, we compare the performance of *subject*, *wilcox* and *mixed* to detect cell subtype markers of CD66+ and CD66- basal cells with bulk RNA-seq data from corresponding PCTs. To avoid confounding the results by disease, this analysis is confined to data from six healthy subjects in the dataset.

**Fig. 5.**
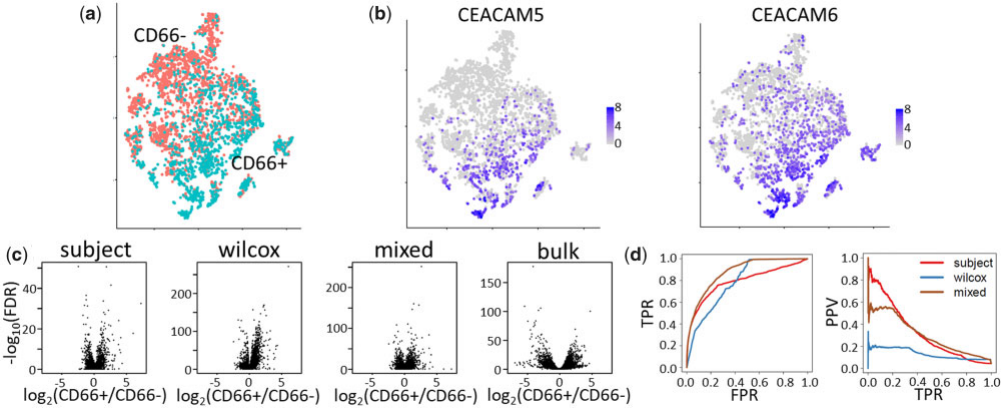
Comparison of methods for detection of CD66+ and CD66- basal cell markers from human trachea. (**a**) t-SNE plot shows CD66+ (turquoise) and CD66- (salmon) basal cells from single-cell RNA-seq profiling of human trachea. (**b**) CD66+ basal cells were identified via detection of CEACAM5 or CEACAM6. (**c**) Volcano plots show results of three methods (subject, wilcox and mixed) used to identify CD66+ and CD66- basal cell marker genes. As a gold standard, results from bulk RNA-seq comparing CD66+ and CD66- basal cells (bulk). (**d**) ROC and PR curves for subject, wilcox and mixed methods using bulk RNA-seq as a gold standard. True positives were identified as those genes in the bulk RNA-seq analysis with FDR < 0.05 and |log_2_(CD66+/CD66–)|>1

Figure 5 shows the results of the marker detection analysis. Compared to the T cell and macrophage marker detection analysis in Section 3.4, we note that the CD66+ and CD66-basal cells are not as transcriptionally distinct (Fig. 5a). The volcano plots for the three scRNA-seq methods have similar shapes, but the *wilcox* and *mixed* methods have inflated adjusted *P*-values relative to *subject* (Fig. 5c).

Figure 5d shows ROC and PR curves for the three scRNA-seq methods using the bulk RNA-seq as a gold standard. In the bulk RNA-seq, genes with adjusted *P*-values less than 0.05 and at least a 2-fold difference in gene expression between CD66+ and CD66-basal cells are considered true positives and all others are considered true negatives. Supplementary Table S1 shows performance measures derived from these curves. In terms of identifying the true positives, *wilcox* and *mixed* had better performance (TPR = 0.62 and 0.56, respectively) than *subject* (TPR = 0.34). On the other hand, *subject* had the smallest FPR (0.03) compared to *wilcox* and *mixed* (0.26 and 0.08, respectively) and had a higher PPV (0.38 compared to 0.10 and 0.23). Overall, *mixed* seems to have the best performance, with a good tradeoff between false positive and TPRs.

### 3.6 DS analysis of healthy and fibrotic alveolar type II cells and alveolar macrophages from human lung

We evaluated the performance of our tested approaches for human multi-subject DS analysis in health and disease. Reyfman *et al.* (2019) used scRNA-seq to profile cells from the lungs of healthy subjects and those with pulmonary fibrosis disease subtypes, including hypersensitivity pneumonitis, systemic sclerosis-associated and myositis-associated interstitial lung diseases and IPF (Reyfman *et al.*, 2019). Further, they used flow cytometry to isolate alveolar type II (AT2) cell and alveolar macrophage (AM) fractions from the lung samples and profiled these PCTs using bulk RNA-seq. We compared the performances of *subject*, *wilcox* and *mixed* for DS analysis of the scRNA-seq from healthy and IPF subjects within AT2 and AM cells using bulk RNA-seq of purified AT2 and AM cell type fractions as a gold standard, similar to the method used in Section 3.5.

**Fig. 6.**
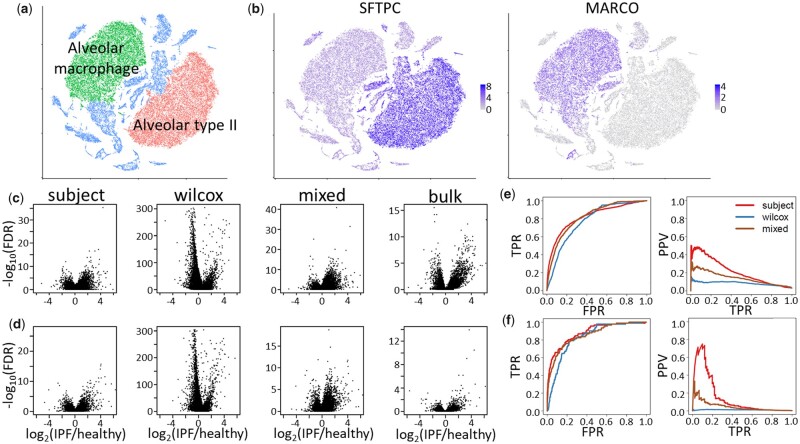
Analysis of AT2 cells and AMs from healthy and IPF lungs. (**a**) t-SNE plot shows AT2 cells (red) and AM (green) from single-cell RNA-seq profiling of human lung from healthy subjects and subjects with IPF. (**b**) AT2 cells and AM express SFTPC and MARCO, respectively. (**c** and **d**) Volcano plots show results of three methods (subject, wilcox and mixed) used to find differentially expressed genes between IPF and healthy lungs in (c) AT2 cells and (d) AM. As a gold standard, results from bulk RNA-seq of isolated AT2 cells and AM comparing IPF and healthy lungs (bulk). (**e** and **f**) ROC and PR curves for subject, wilcox and mixed methods using bulk RNA-seq as a gold standard for (e) AT2 cells and (f) AM. True positives were identified as those genes in the bulk RNA-seq analysis with FDR < 0.05 and |log2(IPF/healthy)|>1

The results of our comparisons are shown in Figure 6. First, we identified the AT2 and AM cells via clustering (Fig. 6a) and plotting well-known markers of these two cell types (Fig. 6b). Next, we used *subject*, *wilcox* and *mixed* to test for differences in expression between healthy and IPF subjects within the AT2 and AM cell populations. Overall, the volcano plots for *subject* and *mixed* look similar with a higher number of genes upregulated in the IPF group, while the *wilcox* method exhibits a much different shape with more genes highly downregulated in the IPF group. Figure 6(e and f) shows ROC and PR curves for the three scRNA-seq methods using the bulk RNA-seq as a gold standard. As in Section 3.5, in the bulk RNA-seq, genes with adjusted *P*-values less than 0.05 and at least a 2-fold difference in gene expression between healthy and IPF are considered true positives and all others are considered true negatives. Supplementary Table S2 contains performance measures derived from the ROC and PR curves. For the AT2 cells (Fig. 6e), *subject* and *mixed* have the same area under the ROC curve (0.82) while the *wilcox* method has slightly smaller area (0.78). Further, *subject* has the highest AUPR (0.21) followed by *mixed* (0.14) and *wilcox* (0.08). For the AM cells (Fig. 6f), the results are similar to AT2 cells with *subject* having the highest areas under the ROC and PR curves (0.88 and 0.15, respectively), followed by *mixed* (0.86 and 0.05, respectively) and *wilcox* (0.83 and 0.01, respectively). The *subject* method has the strongest type I error rate control and highest PPVs, *wilcox* has the highest TPRs and *mixed* has intermediate performance with better TPRs than subject yet lower FPRs than *wilcox* (Supplementary Table S2).

## 4 Discussion

In summary, here we (i) suggested a modeling framework for scRNA-seq data from multiple biological sources, (ii) showed how failing to account for biological variation could inflate the FDR of DS analysis and (iii) provided a formal justification for the validity of ‘pseudobulking’ to allow DS analysis to be performed on scRNA-seq data using software designed for DS analysis of bulk RNA-seq data (Crowell *et al.*, 2020; Lun *et al.*, 2016; McCarthy *et al.*, 2017). Pseudobulking has been tested in real scRNA-seq studies (Kang *et al.*, 2018) and benchmarked extensively via simulation (Crowell *et al.*, 2020). Because pseudobulk methods operate on gene-by-cell count matrices, they are broadly applicable to various single-cell technologies. It is important to emphasize that the aggregation of counts occurs within cell types or cell states, so that the advantages of single-cell sequencing are retained.

As scRNA-seq studies grow in scope, due to technological advances making these studies both less labor-intensive and less expensive, biological replication will become the norm. Further, applying computational methods that account for all sources of variation will be necessary to gain better insights into biological systems, operating at the granular level of cells all the way up to the level of populations of subjects. The analyses presented here have illustrated how different results could be obtained when data were analysed using different units of analysis.

Whereas the pseudobulk method is a simple approach to DS analysis, it has limitations. First, it is assumed that prerequisite steps in the bioinformatic pipeline produced cells that conform to the assumptions of the proposed model. As a counterexample, suppose cells were misclassified, such that cells classified as type A are in reality, composed of a mixture of cells of types A and B. If subjects are composed of different proportions of types A and B, DS results could be due to different cell compositions rather than different mean expression levels.

Second, there may be imbalances in the numbers of cells collected from different subjects. In a study in which a treatment has the effect of altering the composition of cells, subjects in the treatment and control groups may have different numbers of cells of each cell type. Under normal circumstances, the DS analysis should remain valid because the pseudobulk method accounts for this imbalance via different size factors for each subject. In extreme cases, where only a few cells have been collected for some subjects, interpretation of gene expression differences should be handled with caution. This issue is most likely to arise with rare cell types, in which few or no cells are profiled for any subject. In practice, we have omitted comparisons of gene expression in rare cell types because the gene expression profiles had high variation, and the reliability of the comparisons was questionable.

Third, the proposed model also ignores many aspects of the gene expression distribution in favor of simplicity. For example, consider a hypothetical gene having heterogeneous expression in CF pigs, where cells were either ‘low expressors’ or ‘high expressors’ versus homogeneous expression in non-CF pigs, where cells were ‘moderate expressors’. In that case, the number of modes in the expression distribution in the CF group (bimodal) and the non-CF group (unimodal) would be different, but the pseudobulk method may not detect a difference, because it is only able to detect differences in mean expression. A richer model might assume cell-level expression is drawn from a non-parametric family of distributions in the second stage of the proposed model rather than a gamma family.

Improvements in type I and type II error rate control of the DS test could be considered by modeling cell-level gene expression adjusted for potential differences in gene expression between subjects, similar to the *mixed* method in Section 3. The study by Zimmerman *et al.* provides an argument for using mixed models over pseudobulk methods because pseudobulk methods discovered fewer differentially expressed genes. In our simulation study, we also found that the pseudobulk method was conservative, but in some settings, mixed models had inflated FDR. A more powerful statistical test that yields well-controlled FDR could be constructed by considering techniques that estimate all parameters of the hierarchical model. More conventional statistical techniques for hierarchical models, such as maximum likelihood or Bayesian maximum *a posteriori* estimation, could produce less noisy parameter estimates and hence, lead to a more powerful DS test (Gelman and Hill, 2007). These approaches will likely yield better type I and type II error rate control, but as we saw for the *mixed* method in our simulation, the computation times can be substantially longer and the computational burden of these methods scale with the number of cells, whereas the pseudobulk method scales with the number of subjects. Future work with mixed models for scRNA-seq data should focus on maintaining scalable and computationally efficient implementation in software.

Our study highlights user-friendly approaches for analysis of scRNA-seq data from multiple biological replicates. These analyses provide guidance on strengths and weaknesses of different methods in practice. Generally, tests for marker detection, such as the *wilcox* method, are sufficient if type I error rate control is less of a concern than type II error rate and in circumstances where type I error rate is most important, methods like *subject* and *mixed* can be used. Until computationally efficient methods exist to fit hierarchical models incorporating all sources of biological variation inherent to scRNA-seq, we believe that pseudobulk methods are useful tools for obtaining time-efficient DS results with well-controlled FDR.

## Data availability

The data from pig airway epithelia underlying this article are available in GEO and can be accessed with GEO accession GSE150211. Data for the analysis of human skin biopsies were obtained from GEO accession GSE130973. Data for the analysis of human trachea were obtained from GEO accessions GSE143705 (bulk RNA-seq) and GSE143706 (scRNA-seq). The scRNA-seq data for the analysis of human lung tissue were obtained from GEO accession GSE122960, and the bulk RNA-seq of purified AT2 and AM fractions were shared by the authors immediately upon request.

## Supplementary Material

btab337_supplementary_data
