# Supplementary material for: Differential gene expression analysis for multi-subject single-cell RNA-sequencing studies with aggregateBioVar
Source: Bioinformatics. 2021 May 10;37(19):3243–51. doi: 10.1093/bioinformatics/btab337 (PMC8504643; doi:10.1093/bioinformatics/btab337)
Supplement: btab337_supplementary_data [file btab337_supplementary_data.docx]

**Supplementary text: example code for *aggregateBioVar***

library(SummarizedExperiment)

library(muscat)

library(scater)

library(aggregateBioVar)

aggregateBioVar provides a simple implementation of subject-level aggregation for count sums by cell type. Here, a list is returned with a SummarizedExperiment object (Huber et al. 2015) for each aggregate cell type from an input SingleCellExperiment (Amezquita et al. 2020). Two other implementations for count aggregation are provided by the Bioconductor packages scater (McCarthy et al. 2017) and muscat (Crowell et al. 2020). The former retains aggregate counts in a single assay slot of a SingleCellExperiment object. From there, subject level aggregates for a given cell type can be accessed by subsetting the column metadata. The latter creates a new assay slot for each cell type aggregate by subject.

aggregateBioVar(

scExp = small_airway,

subjectVar = "orig.ident",

cellVar = "celltype"

)

## Coercing metadata variable to character: celltype

## $AllCells

## class: SummarizedExperiment

## dim: 1339 7

## metadata(0):

## assays(1): counts

## rownames(1339): MPC1 PRKN ... OTOP1 UNC80

## rowData names(0):

## colnames(7): SWT1 SWT2 ... SWT4 SCF3

## colData names(3): orig.ident Genotype Region

##

## $`Immune cell`

## class: SummarizedExperiment

## dim: 1339 7

## metadata(0):

## assays(1): counts

## rownames(1339): MPC1 PRKN ... OTOP1 UNC80

## rowData names(0):

## colnames(7): SWT1 SWT2 ... SWT4 SCF3

## colData names(4): orig.ident Genotype Region celltype

##

## $`Secretory cell`

## class: SummarizedExperiment

## dim: 1339 7

## metadata(0):

## assays(1): counts

## rownames(1339): MPC1 PRKN ... OTOP1 UNC80

## rowData names(0):

## colnames(7): SWT1 SWT2 ... SWT4 SCF3

## colData names(4): orig.ident Genotype Region celltype

##

## $`Endothelial cell`

## class: SummarizedExperiment

## dim: 1339 7

## metadata(0):

## assays(1): counts

## rownames(1339): MPC1 PRKN ... OTOP1 UNC80

## rowData names(0):

## colnames(7): SWT1 SWT2 ... SWT4 SCF3

## colData names(4): orig.ident Genotype Region celltype

- muscat creates a new assay slot for each cell type aggregate by subject

muscat_aggregate <-

muscat::aggregateData(

x = small_airway, assay = "counts",

fun = 'sum', by = c("celltype", "orig.ident")

)

dim(muscat_aggregate)

## [1] 1339 7

assays(muscat_aggregate)

## List of length 3

## names(3): Endothelial cell Immune cell Secretory cell

- scater extends the assay slot for each cell type by subject

scater_aggregate <-

scuttle::aggregateAcrossCells(

x = small_airway,

id = colData(small_airway)[, c("celltype", "orig.ident")]

)

dim(scater_aggregate)

## [1] 1339 21

scater_aggregate$celltype

## [1] Endothelial cell Endothelial cell Endothelial cell Endothelial cell

## [5] Endothelial cell Endothelial cell Endothelial cell Immune cell

## [9] Immune cell Immune cell Immune cell Immune cell

## [13] Immune cell Immune cell Secretory cell Secretory cell

## [17] Secretory cell Secretory cell Secretory cell Secretory cell

## [21] Secretory cell

## Levels: Endothelial cell Immune cell Secretory cell

**Supplementary Figures**

**
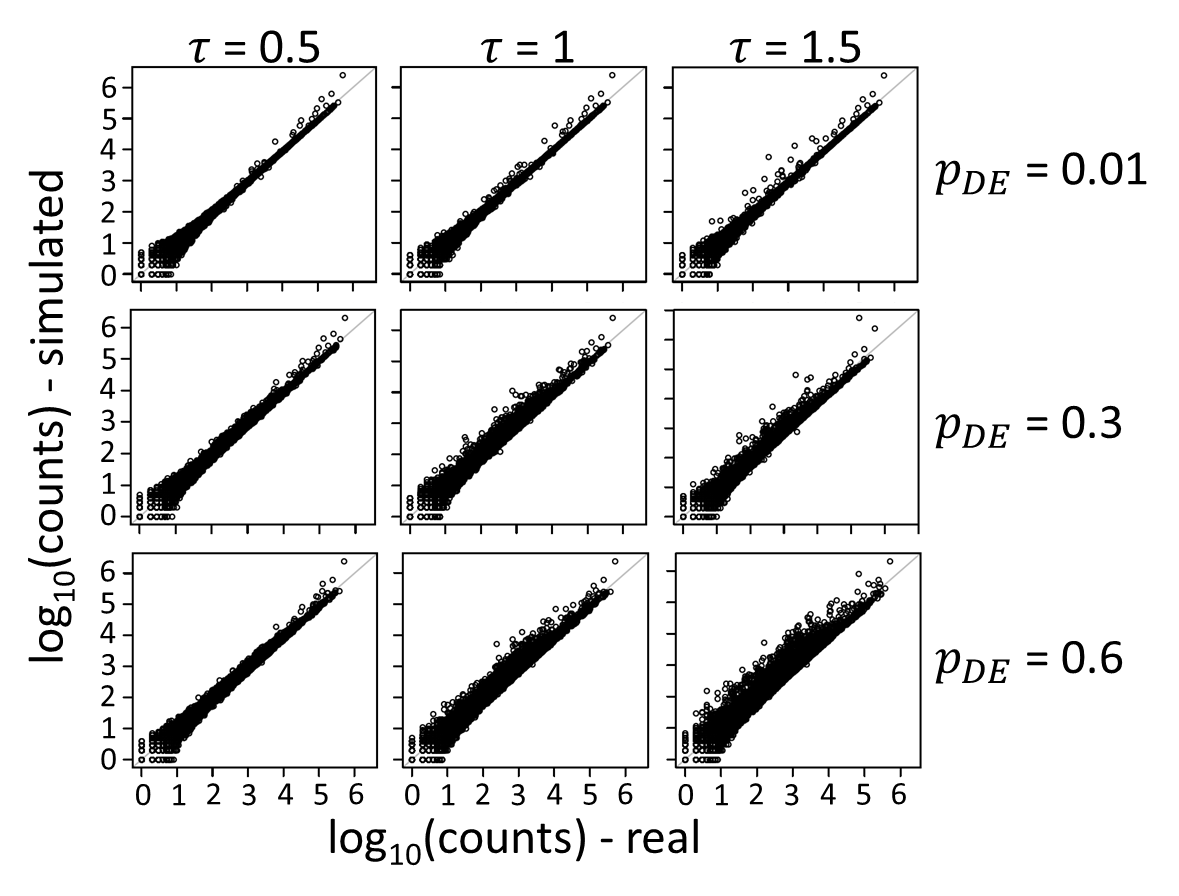
**

**Supplementary Figure 1. Concordance between simulated and real data:** Each panel shows the agreement between simulated and real data counts for one simulation setting. The counts for each gene were summed across all cells and log_10_-transformed. Rows correspond to different proportions of differentially expressed genes, $p_{DE}$, and columns correspond to different standard deviations of (natural) log fold change, $\tau$. The vertical axis gives the log_10_-transformed counts for the simulated data, and the horizontal axis gives the log_10_-transformed counts for the real data.


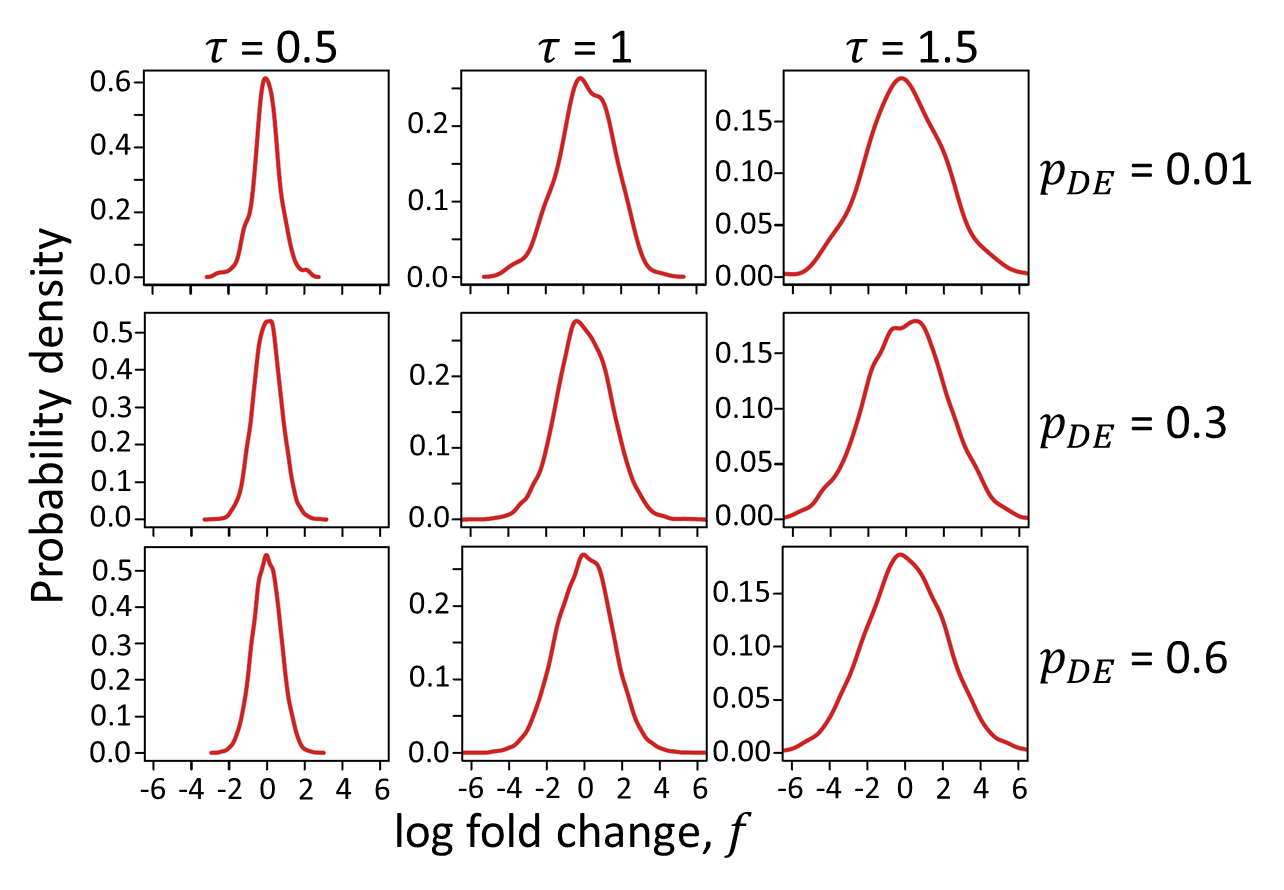


**Supplementary Figure 2. Distribution of log fold change in simulated data** Each panel shows a kernel estimate of the probability density of (natural) log fold change between groups 1 and 2 for one simulation setting. Rows correspond to different proportions of differentially expressed genes, $p_{DE}$, and columns correspond to different standard deviations of (natural) log fold change, $\tau$. The horizontal axis gives the value of log fold change, $f$, and the vertical axis gives the probability density at $f$.


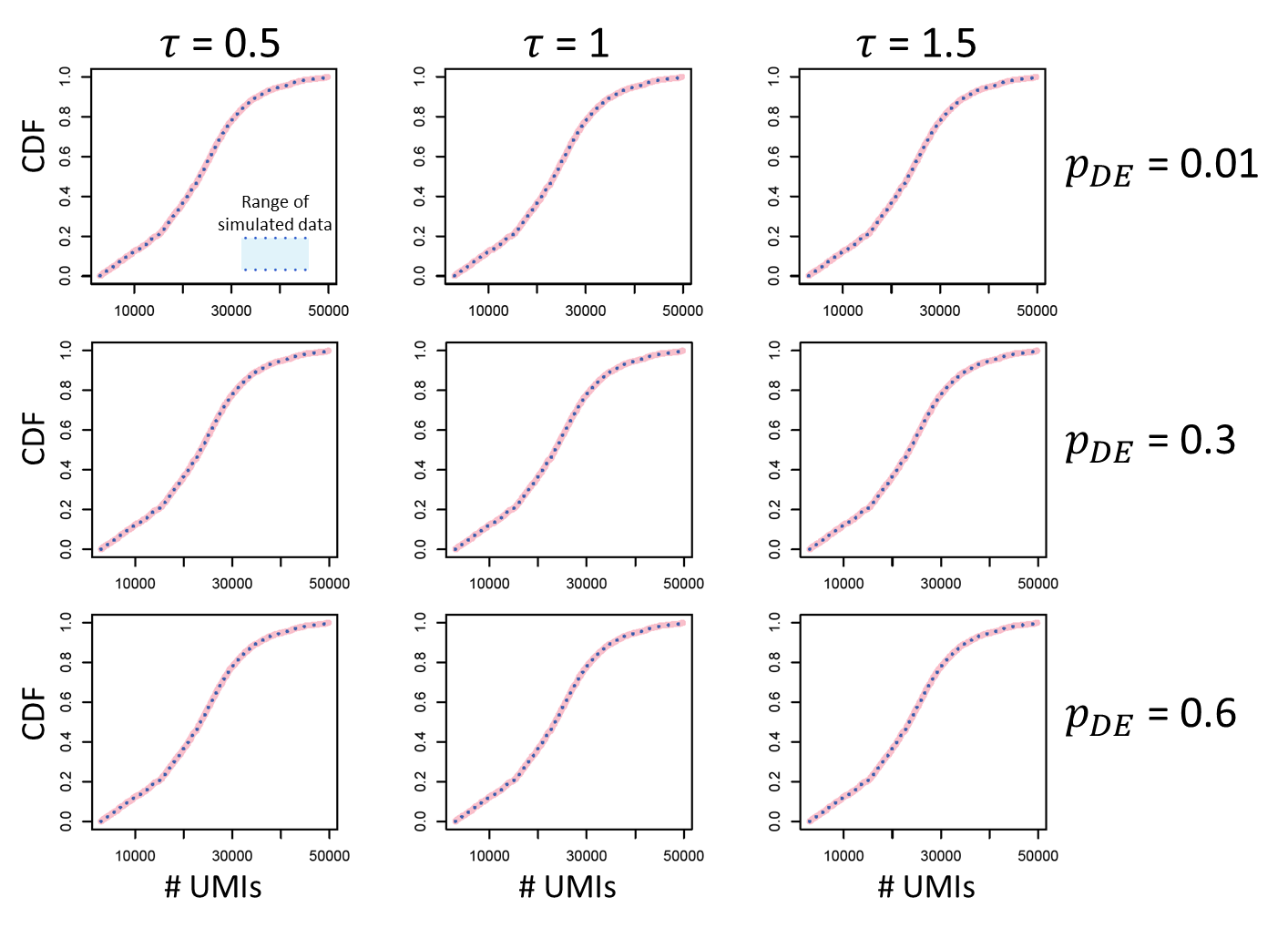


**Supplementary Figure 3. Number of UMIs in simulated and real data** Each panel shows the distribution of number of UMIs per cell (as cumulative distribution function) in the real data set (pink curve). Dotted blue lines give the range of cumulative distribution function values in the 100 simulated data sets. Rows correspond to different proportions of differentially expressed genes, $p_{DE}$, and columns correspond to different standard deviations of (natural) log fold change, $\tau$.


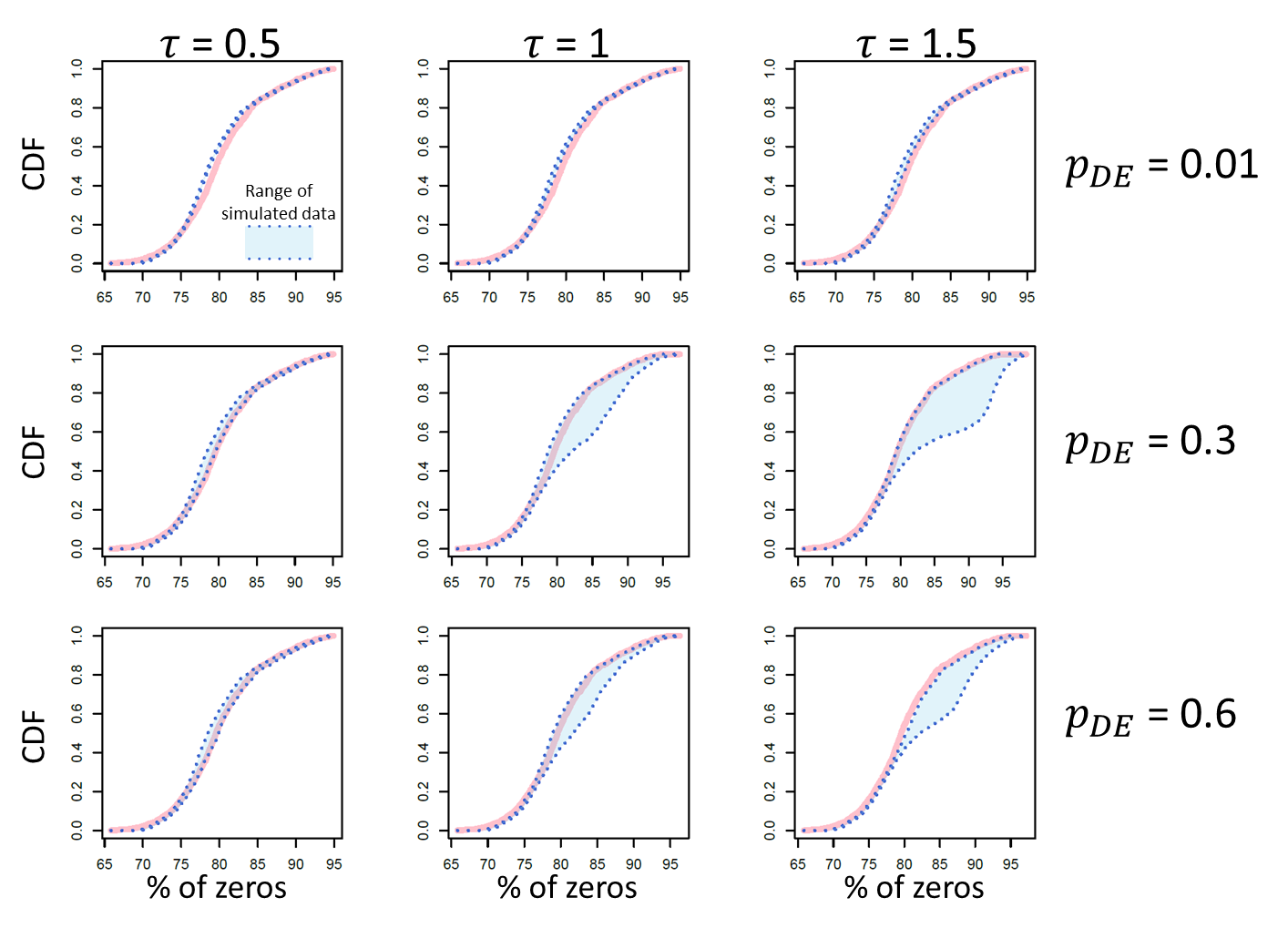


**Supplementary Figure 4.** **Percentage of zero counts per cell in simulated and real data** Each panel shows the distribution of percentage of zero counts per cell (as cumulative distribution function) in the real data set (pink curve). Dotted blue lines give the range of cumulative distribution function values in the 100 simulated data sets. Rows correspond to different proportions of differentially expressed genes, $p_{DE}$, and columns correspond to different standard deviations of (natural) log fold change, $\tau$.


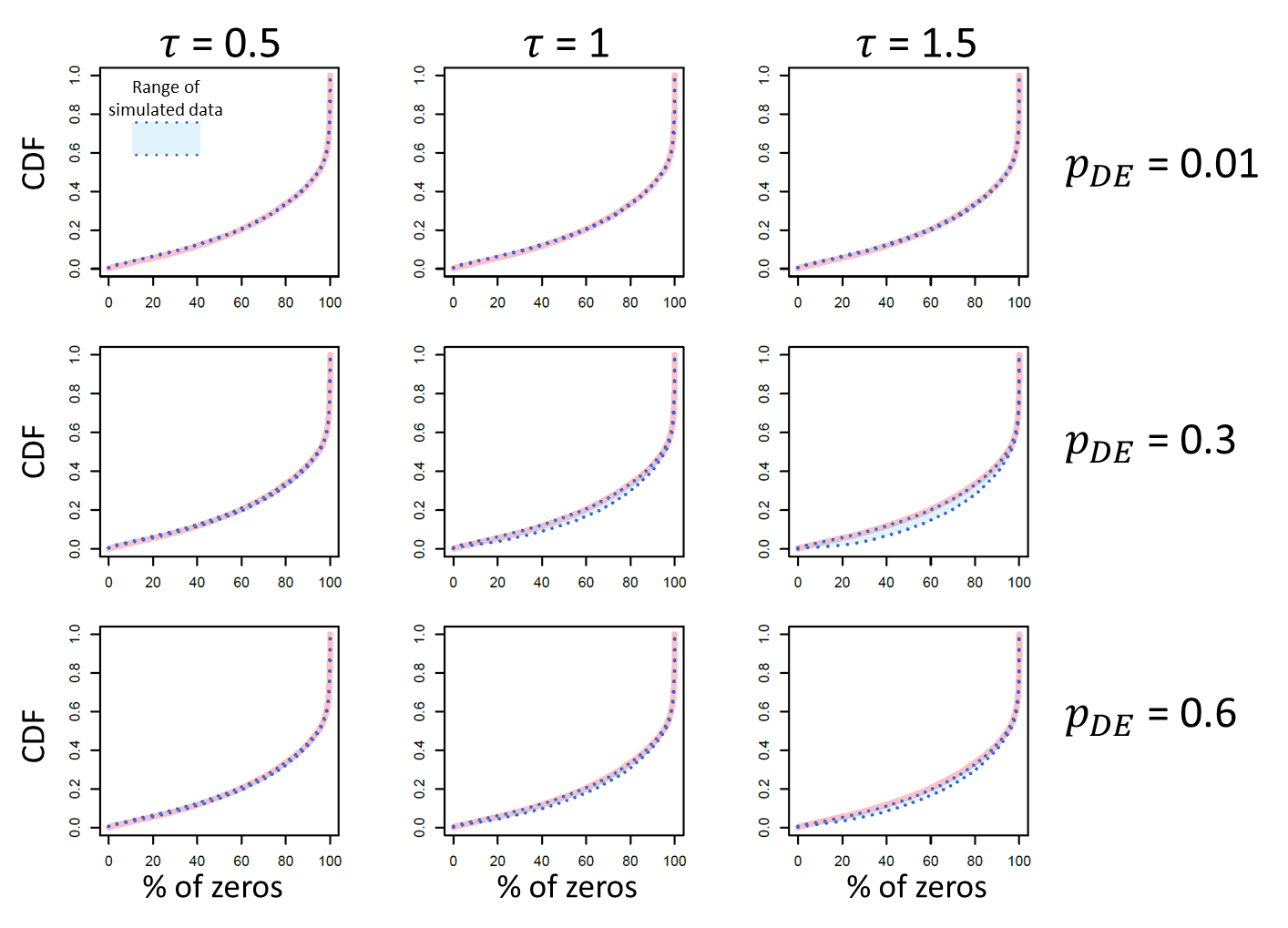


**Supplementary Figure 5. Percentage of zero counts per gene in simulated and real data** Each panel shows the distribution of percentage of zero counts per gene (as cumulative distribution function) in the real data set (pink curve). Dotted blue lines give the range of cumulative distribution function values in the 100 simulated data sets. Rows correspond to different proportions of differentially expressed genes, $p_{DE}$, and columns correspond to different standard deviations of (natural) log fold change, $\tau$.


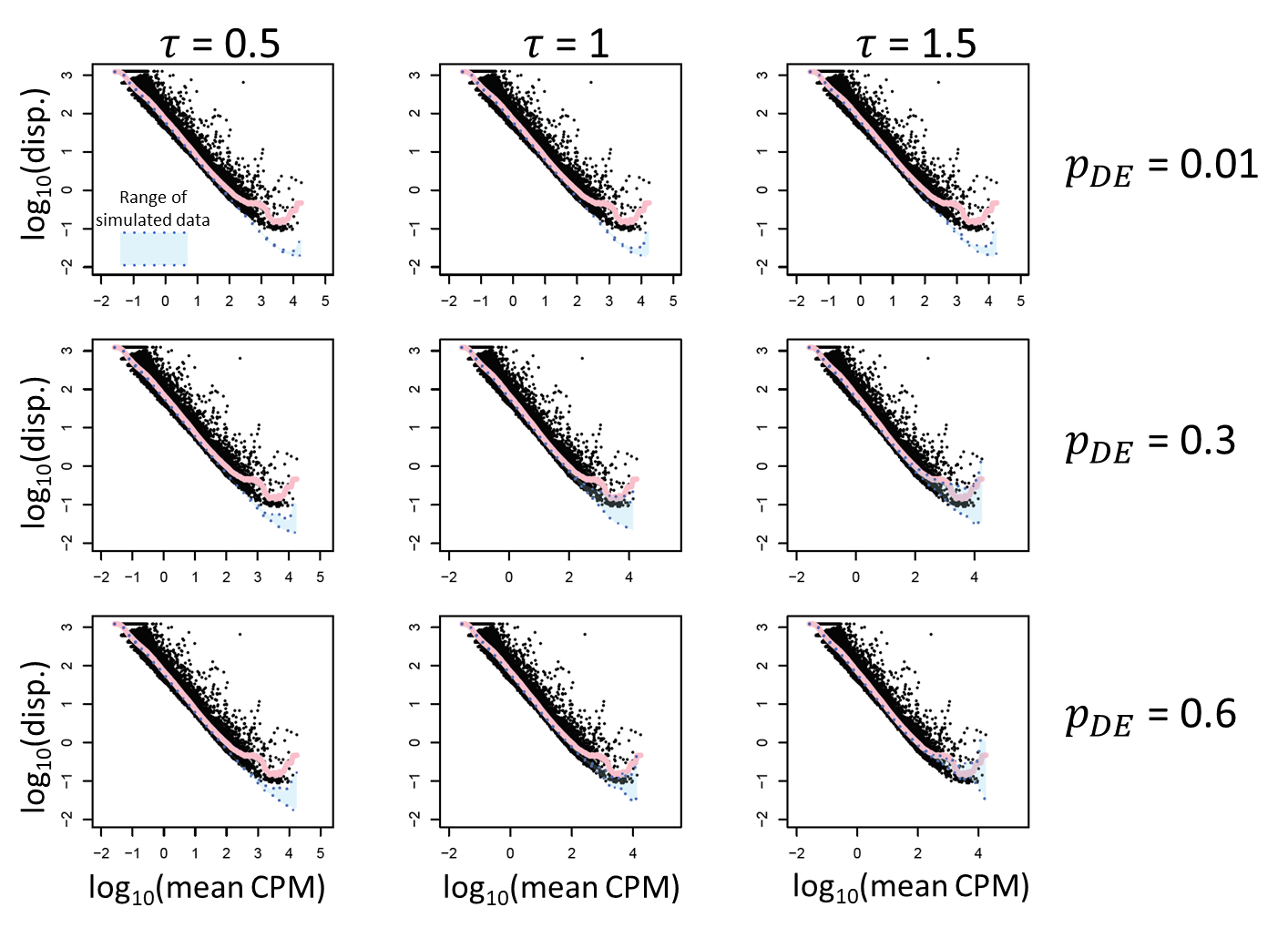


**Supplementary Figure 6. Mean-dispersion relationship in simulated and real data** Each panel shows the relationship between dispersion and mean expression. Dispersion was defined as variance of gene expression (in counts per million) divided by squared mean of gene expression (in counts per million). Each black dot is a gene in the real data set, and the pink curve is a kernel regression estimate of the relationship. Dotted blue lines give the range of kernel regression estimates in the 100 simulated data sets. Rows correspond to different proportions of differentially expressed genes, $p_{DE}$, and columns correspond to different standard deviations of (natural) log fold change, $\tau$.

**
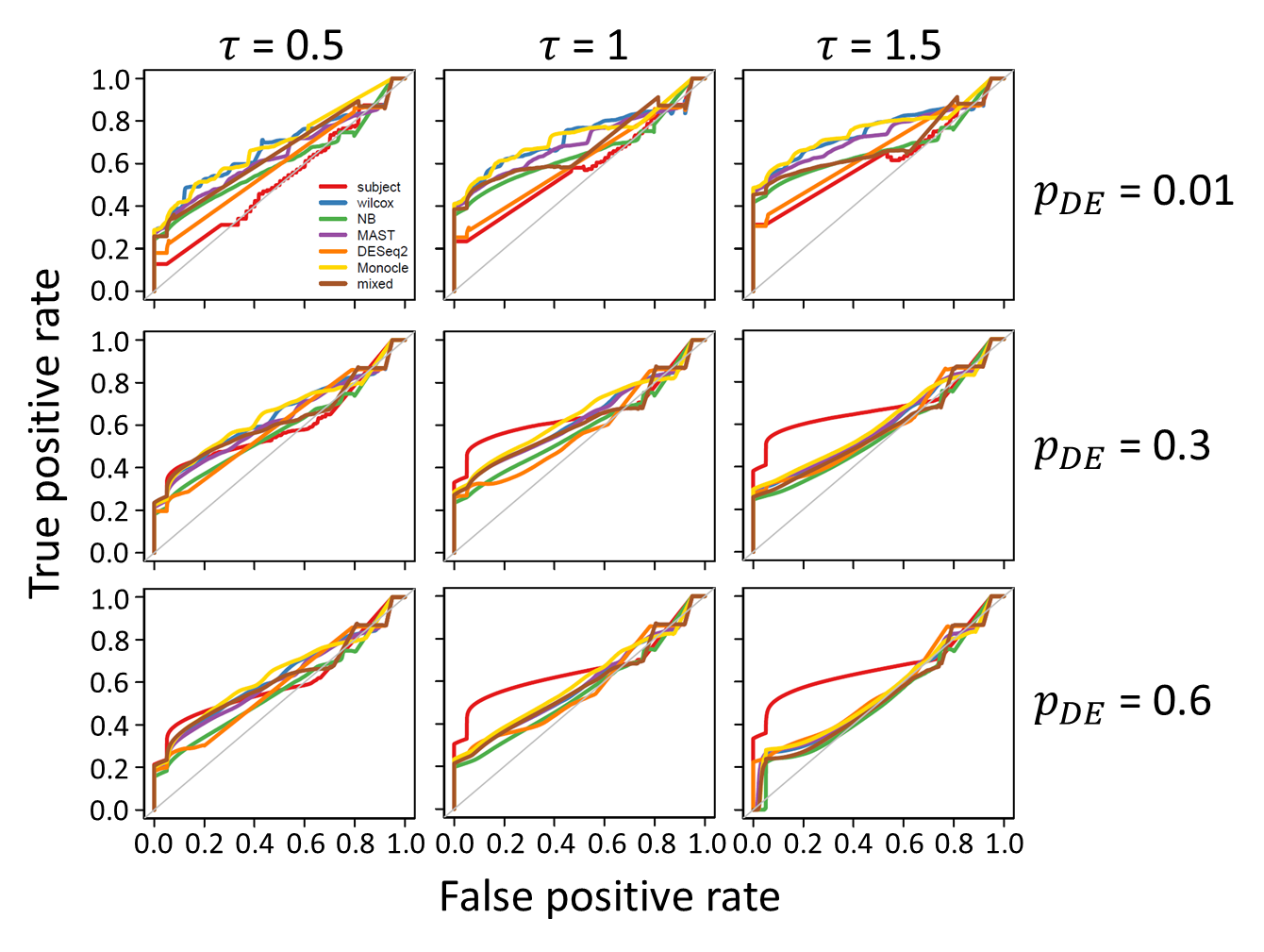
**

**Supplementary Figure 7. Receiver operating characteristic (ROC) curves for differential state analysis methods** Each panel shows mean ROC curves for 100 simulated data sets in one simulation setting. Rows correspond to different proportions of differentially expressed genes, $p_{DE}$, and columns correspond to different standard deviations of (natural) log fold change, $\tau$. In each panel, ROC curves are plotted for each of seven differential state analysis methods: subject (red), wilcox (blue), NB (green), MAST (purple), DESeq2 (orange), Monocle (gold), and mixed (brown). The vertical axis gives the true positive rate, and the horizontal axis gives false positive rate.

**
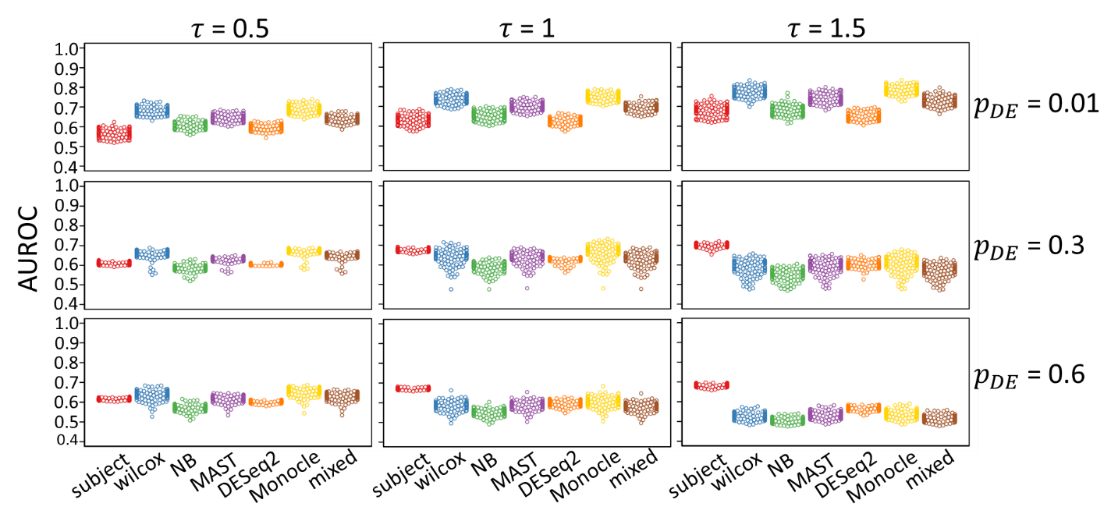
**

**
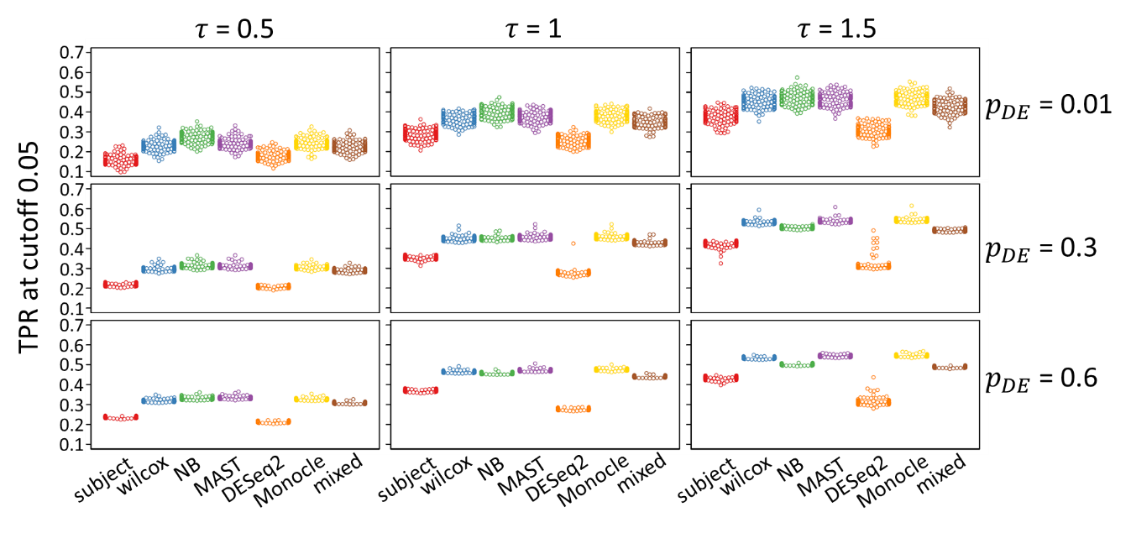
**

**
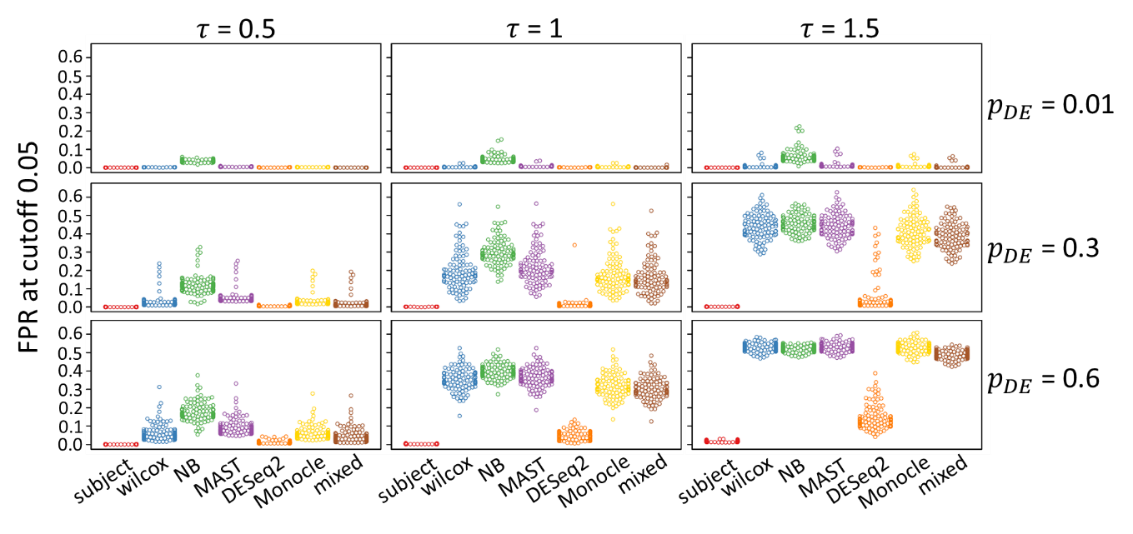
**

**Supplementary Figure 8. Additional performance measures for differential state analysis of simulated data** (a) Area under the receiver operating characteristic curve (AUROC), (b) true positive rate (TPR) with adjusted p-value cutoff 0.05, and (c) false positive rate (FPR) with adjusted p-value cutoff 0.05 for seven differential state analysis methods. Each panel shows results for 100 simulated data sets in one simulation setting. Rows correspond to different proportions of differentially expressed genes, $p_{DE}$, and columns correspond to different standard deviations of (natural) log fold change, $\tau$. The implemented methods are subject (red), wilcox (blue), NB (green), MAST (purple), DESeq2 (orange), Monocle (gold), and mixed (brown). The vertical axes give the performance measures, and the horizontal axes label each method.


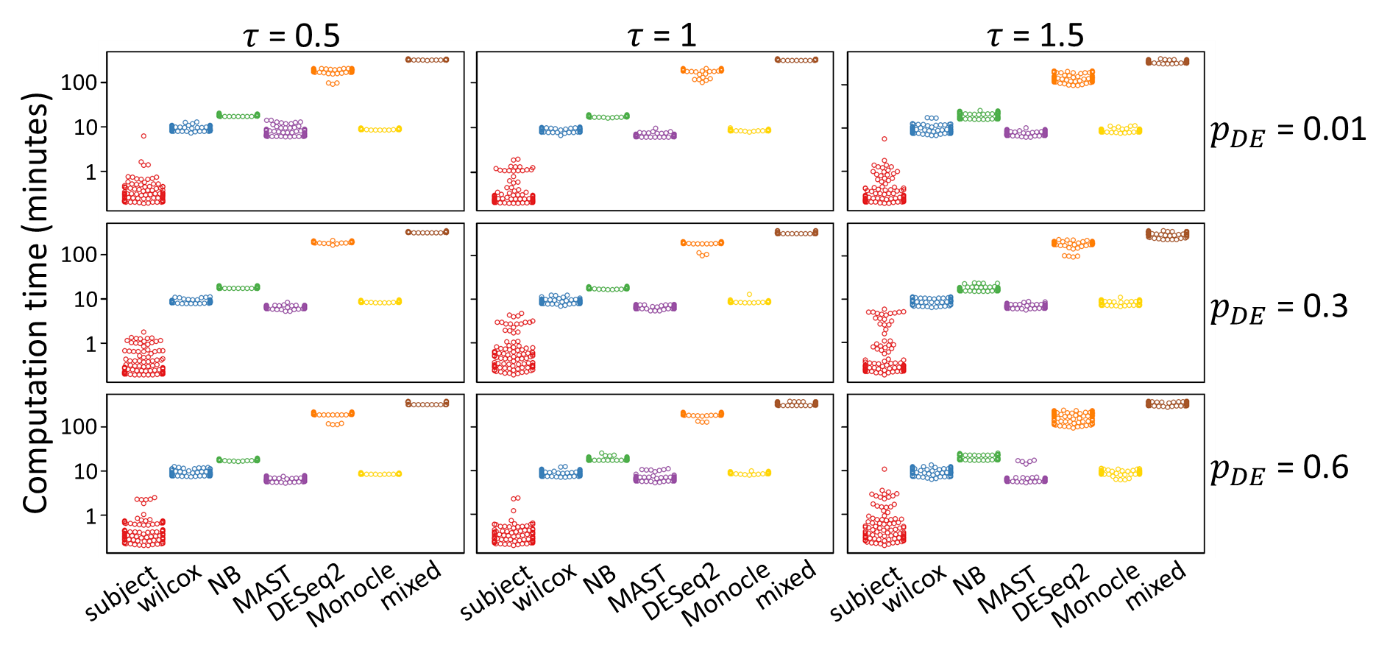


**Supplementary Figure 9. Computation time for differential state analysis of simulated data** Each panel shows computation times for 100 simulated data sets in one simulation setting. Rows correspond to different proportions of differentially expressed genes, $p_{DE}$, and columns correspond to different standard deviations of (natural) log fold change, $\tau$. The implemented methods are subject (red), wilcox (blue), NB (green), MAST (purple), DESeq2 (orange), Monocle (gold), and mixed (brown). The vertical axes give the performance measures, and the horizontal axes label each method.

**
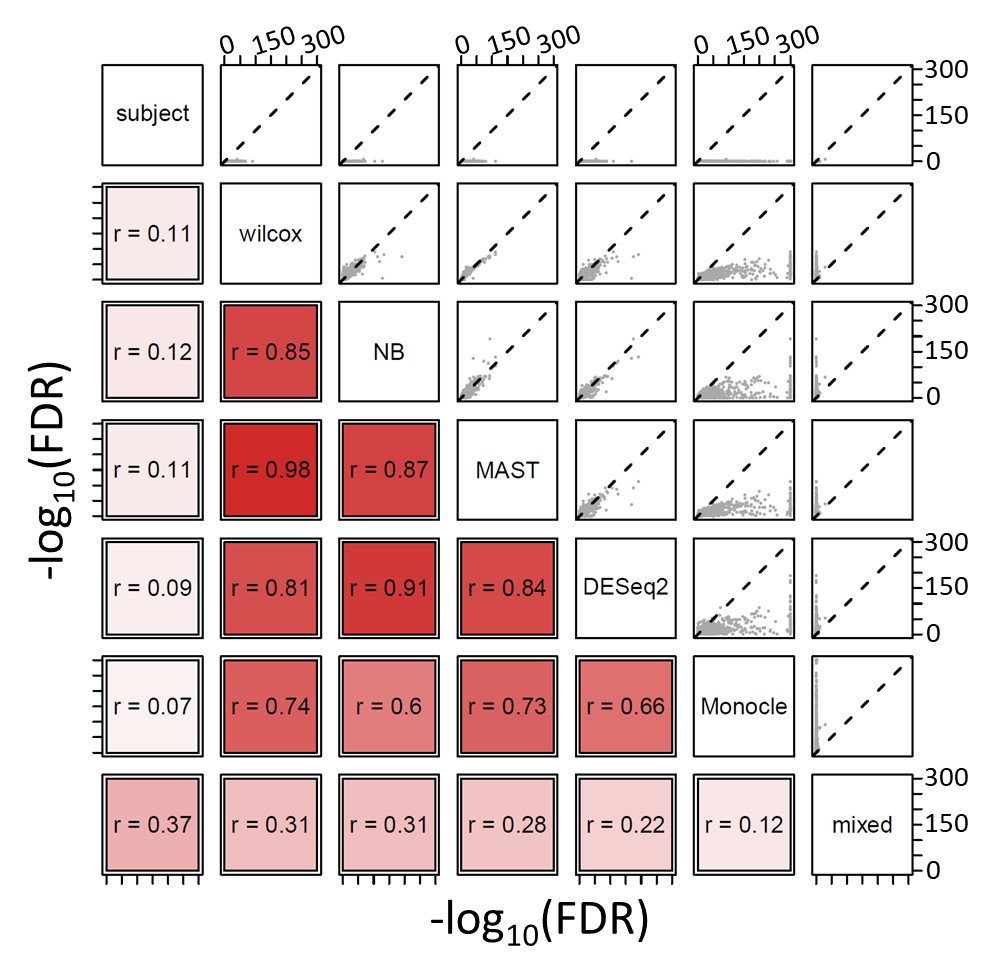
**

**Supplementary Figure 10. Concordance between adjusted p-values for differential state analysis methods in analysis of CF and non-CF small airway secretory cells** For the differential state analysis of pig small airway secretory cells, adjusted p-values (FDR) for each method were negative log_10_-transformed and compared. The diagonal identifies the method corresponding to each row and column, the upper diagonal gives scatterplots of concordance between each pair of methods, and the lower diagonal gives the Pearson correlation between each pair of methods with darker shades of red corresponding to higher concordance between two methods.


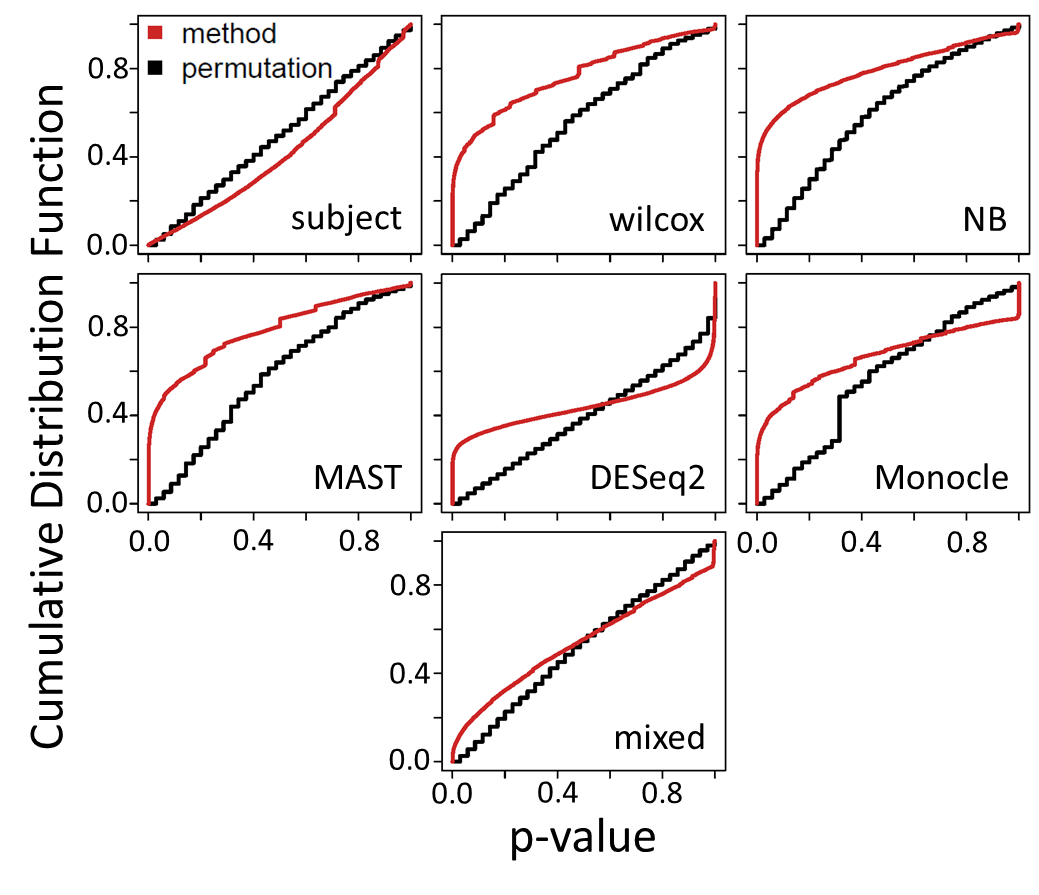


**Supplementary Figure 11. Comparison of method p-values and permutation p-values for analysis of CF and non-CF pig small airway secretory cells** Each panel shows the distributions of method p-values red) and permutation p-values (black) for a different method. Vertical axes give values of the cumulative distribution function at p-values on the horizontal axes.

**
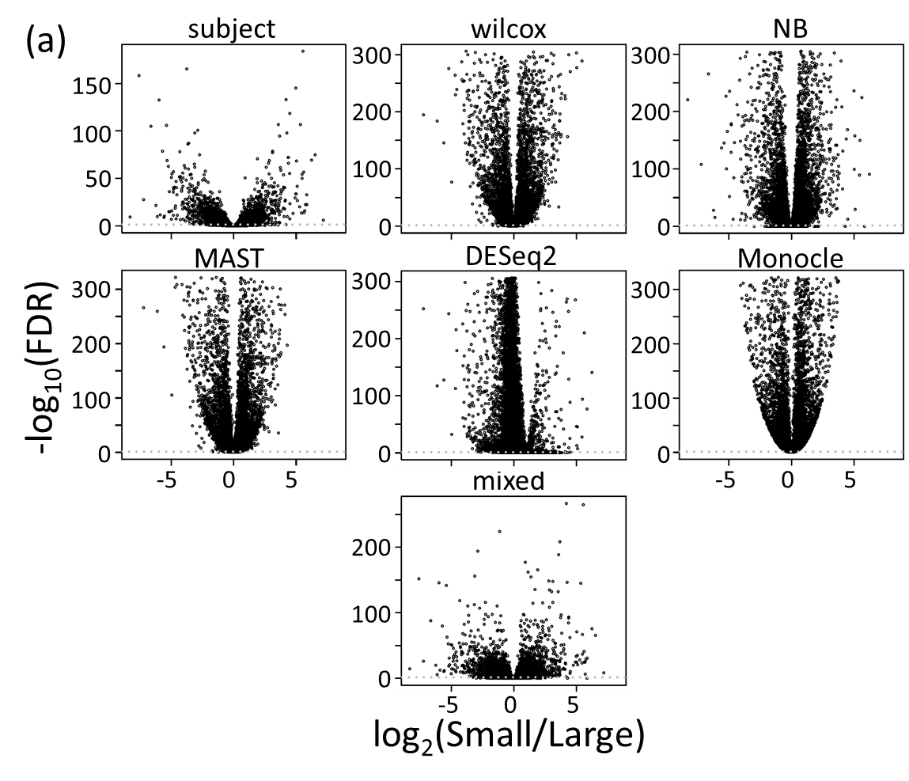
**

**
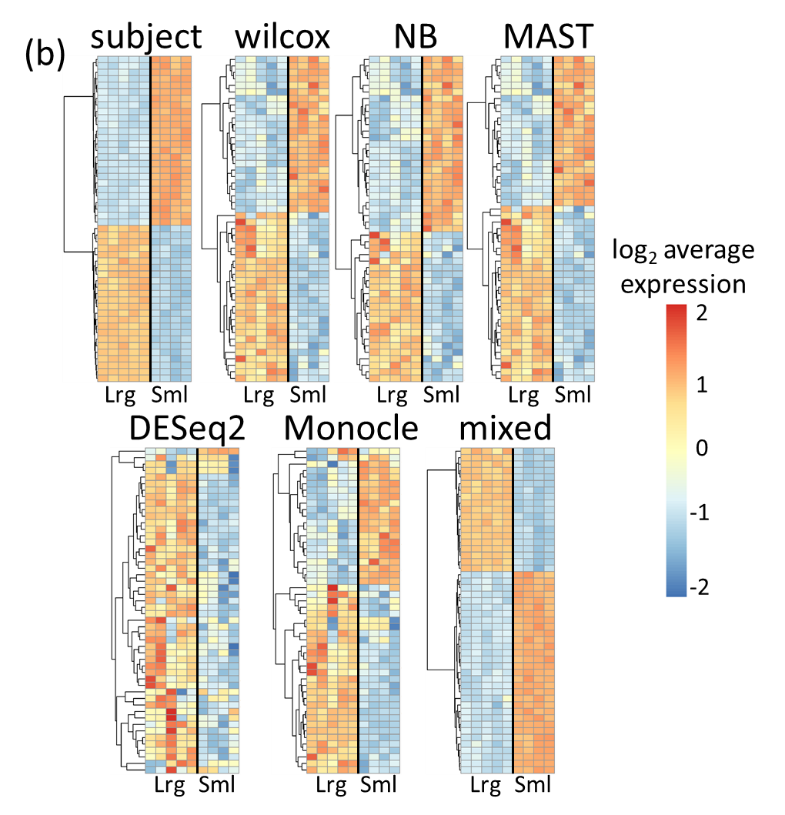
**

**Supplementary Figure 12. Results for analysis of large and small airways in non-CF pig ciliated cells** (a) Volcano plots and (b) heatmaps of top 50 genes for seven different differential state analysis methods. In (a), vertical axes are negative log_10_-transformed adjusted p-values, and horizontal axes are log_2_-transformed fold changes. In (b), rows correspond to different genes, and columns correspond to different pigs. The top 50 genes for each method were defined to be the 50 genes with smallest adjusted p-values. Subject-level gene expression scores were computed as the average counts per million for all cells from each subject. Lrg= large airway; Sml = small airway

**
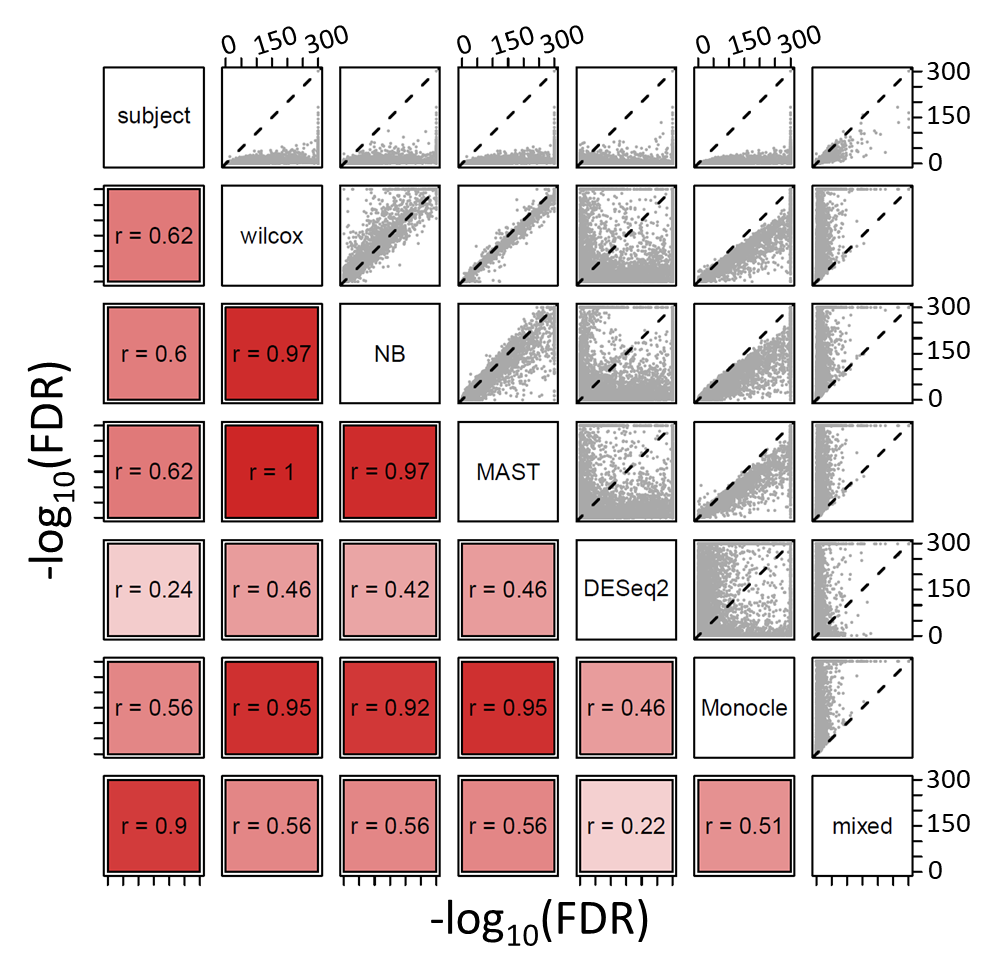
**

**Supplementary Figure 13. Concordance between adjusted p-values for differential state analysis methods in analysis of non-CF small and large airway ciliated cells** For the differential state analysis of ciliated cells from non-CF pigs, adjusted p-values (FDR) for each method were negative log_10_-transformed and compared. The diagonal identifies the method corresponding to each row and column, the upper diagonal gives scatterplots of concordance between each pair of methods, and the lower diagonal gives the Pearson correlation between each pair of methods with darker shades of red corresponding to higher concordance between two methods.


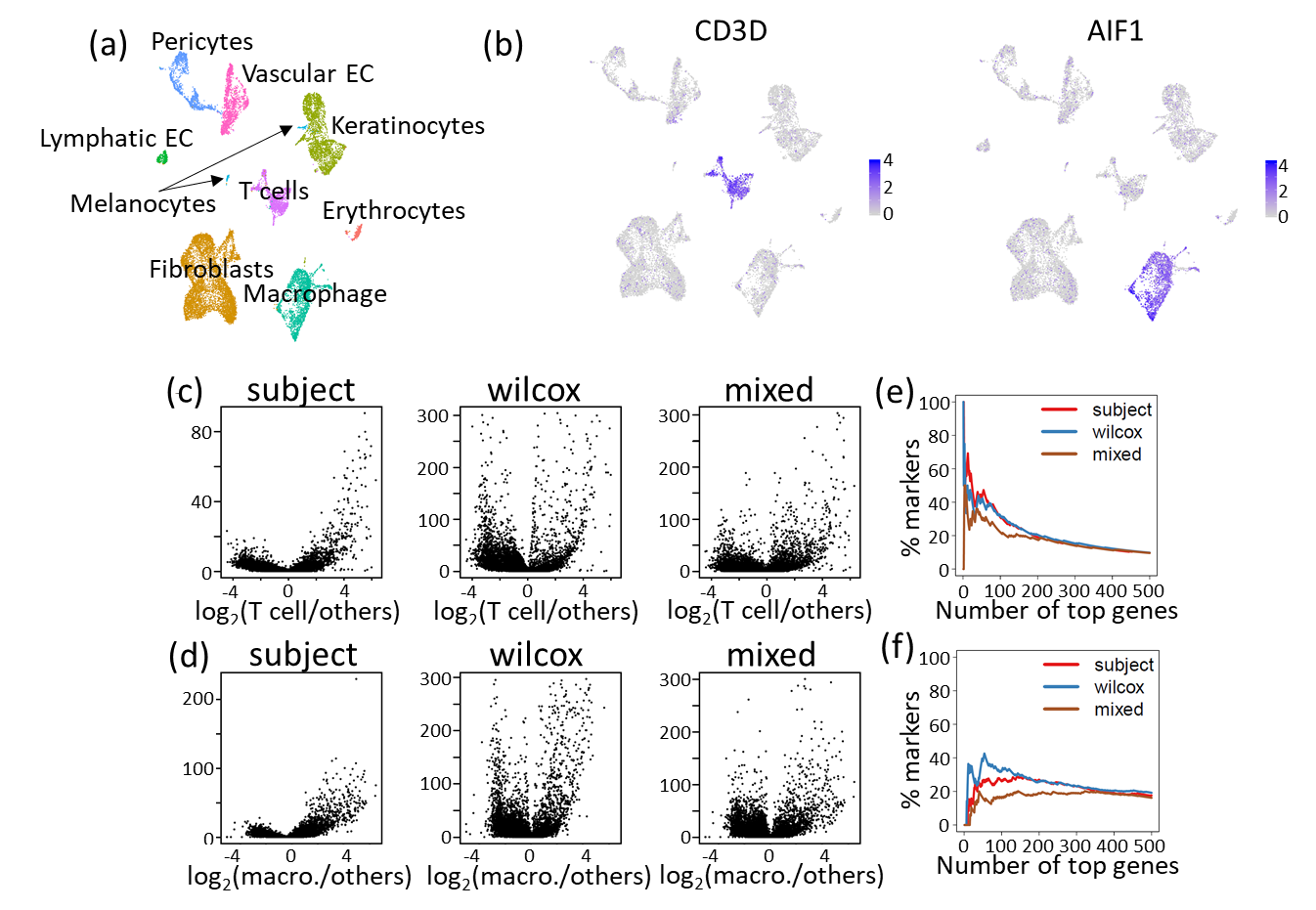
**Supplementary Figure 14.** **Comparison of methods for detection of T cell and macrophage markers** (a) UMAP shows single cell RNA-seq profiling of human skin cells. (b) T cells and macrophages were identified using markers *CD3D* and *AIF1*. (c-d) Volcano plots show results of three methods (subject, wilcox, and mixed) used to identify (c) T cell and (d) macrophage marker genes. (e-f) Among the “top genes” (horizontal axis), the percentage that are known markers (vertical axis) of (e) T cells and (f) macrophages. Top genes were defined by sorting FDR from smallest to largest.

**Supplementary Tables**

**Supplementary Table 1. Performance of three methods for marker detection of CD66+ and CD66- basal cells from human trachea** Operating characteristics were measured for three methods (subject, wilcox, and mixed) used to find markers of CD66+ and CD66- basal cells. As a gold standard, true positives were identified as those genes in bulk RNA-seq analysis with FDR<0.05 and |log_2_(FC)|>1. Performance measures included area under the receiver operating characteristic curve (AUROC), area under the precision-recall curve (AUPR), and four measurements of accuracy using an adjusted p-value cutoff of 0.05: true positive rate (TPR), false positive rate (FPR), positive predictive value (PPV), and negative predictive value (NPV).

**
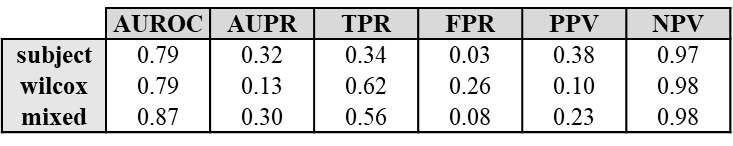
**

**Supplementary Table 2. Performance of three methods for differential expression analysis of alveolar type II (AT2) cells and alveolar macrophages (AM) from healthy and IPF lungs** Operating characteristics were measured for three methods (subject, wilcox, and mixed) used to find differentially expressed genes between IPF and healthy lungs in AT2 cells and AM. As a gold standard, true positives were identified as those genes in bulk RNA-seq analysis with FDR<0.05 and |log_2_(FC)|>1. Performance measures included area under the receiver operating characteristic curve (AUROC), area under the precision-recall curve (AUPR), and four measurements of accuracy using an adjusted p-value cutoff of 0.05: true positive rate (TPR), false positive rate (FPR), positive predictive value (PPV), and negative predictive value (NPV).

**
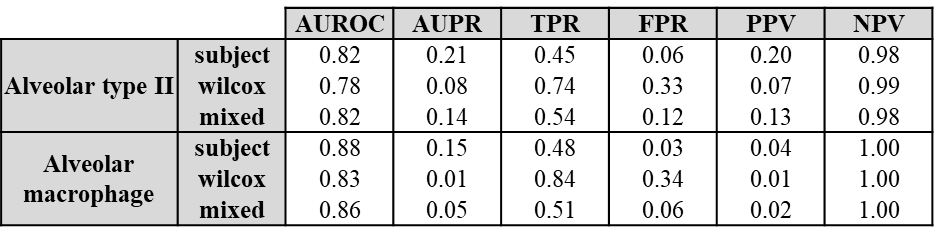
**

**References**

Amezquita, Robert, Aaron Lun, Etienne Becht, Vince Carey, Lindsay Carpp, Ludwig Geistlinger, Federico Marini, et al. 2020. “Orchestrating Single-Cell Analysis with Bioconductor.” Nature Methods 17: 137–45. https://www.nature.com/articles/s41592- 019-0654-x.

Crowell, Helena L., Charlotte Soneson, Pierre-Luc Germain, Daniela Calini, Ludovic Collin, Catarina Raposo, Dheeraj Malhotra, and Mark D. Robinson. 2020. “Muscat Detects Subpopulation-Specific State Transitions from Multi-Sample Multi-Condition Single- Cell Transcriptomics Data.” Nature Communications 11 (1): 6077. https://doi.org/10.1038/s41467-020-19894-4.

Huber, W., V. J. Carey, R. Gentleman, S. Anders, M. Carlson, B. S. Carvalho, H. C. Bravo, et al. 2015. “Orchestrating High-Throughput Genomic Analysis with Bioconductor.” Nature Methods 12 (2): 115–21. http://www.nature.com/nmeth/journal/v12/n2/full/nmeth.3252.html.

McCarthy, Davis J., Kieran R. Campbell, Aaron T. L. Lun, and Quin F. Willis. 2017. “Scater: Pre-Processing, Quality Control, Normalisation and Visualisation of Single-Cell RNA- Seq Data in R.” Bioinformatics 33 (8): 1179–86. https://doi.org/10.1093/bioinformatics/btw777.
